# Nuclear Import Defects Drive Cell Cycle Dysregulation in Neurodegeneration

**DOI:** 10.1111/acel.70091

**Published:** 2025-05-16

**Authors:** Jonathan Plessis‐Belair, Taylor Russo, Markus Riessland, Roger B. Sher

**Affiliations:** ^1^ Department of Neurobiology and Behavior Stony Brook University Stony Brook New York USA; ^2^ Center for Nervous System Disorders Stony Brook University Stony Brook New York USA

**Keywords:** cell cycle, neurodegeneration, nuclear import

## Abstract

Neurodegenerative diseases (NDDs) and other age‐related disorders have been classically defined by a set of key pathological hallmarks. Two of these hallmarks, cell cycle dysregulation (CCD) and nucleocytoplasmic transport (NCT) defects, have long been debated as being either causal or consequential in the pathology of accelerated aging. Specifically, aberrant cell cycle activation in post‐mitotic neurons has been shown to trigger neuronal cell death pathways and cellular senescence. Additionally, NCT has been observed to be progressively dysregulated during aging and in neurodegeneration, where the increased subcellular redistribution of nuclear proteins, such as TAR DNA‐Binding Protein‐43 (TDP‐43), to the cytoplasm is a primary driver of disease. However, the functional significance of NCT defects as either a causal mechanism or consequence of pathology, and how the redistribution of cell cycle machinery contributes to neurodegeneration, remains unclear. Here, we describe that pharmacological inhibition of importin‐β nuclear import is capable of perturbing cell cycle machinery both in mitotic neuronal cell lines and post‐mitotic primary neurons in vitro. Our *Nemf* ^R86S^ mouse model of motor neuron disease, characterized by nuclear import defects, further recapitulates the hallmarks of CCD we observed in mitotic cell lines and in post‐mitotic primary neurons in vitro, and in spinal motor neurons in vivo. The observed CCD is consistent with the transcriptional and phenotypical dysregulation commonly associated with neuronal cell death and senescence‐like features in NDDs. Together, this evidence suggests that impairment of nuclear import pathways resulting in CCD may be a common driver of pathology in neurodegeneration.

## Graphical abstract

Overview of Dysregulated Cell Cycle Mechanisms in Neuronal Cells. A nuclear import block drives cell cycle re‐entry from G_0_, culminating in cell cycle arrest at G_1_/S. This cell cycle arrest is associated with activation of CKIs from the INK locus (p15, p16, p18, p19) and Cip/Kip (p21, p27) which act on specific CDK/Cyclin complexes. This activity is further associated with the G_1_/S downregulation of E2F and stathmins, resulting in microtubule dysregulation and cell cycle arrest.

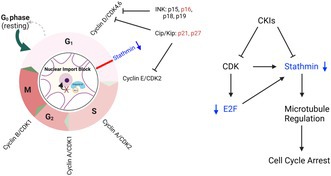

## 1 Introduction

Aging is the primary risk factor for both cancer and neurodegenerative diseases (NDDs) (Hou et al. 2019; Sedrak and Cohen 2023). Both replicative and physiological aging are intimately linked to cell‐cycle decisions, and the ability of cells to undergo cell‐cycle arrest in response to different triggers is a crucial process required for the maintenance of genomic integrity (Pietenpol and Stewart 2002). Whereas cell cycle dysregulation (CCD) has traditionally been associated as a hallmark of tumor cells, the inappropriate activation of cell cycle regulators has also been implicated in the pathogenesis of NDDs (Herrup 2012; Herrup and Arendt 2002; Herrup and Yang 2007; Martínez‐Cué and Rueda 2020; Stewart et al. 2003; Wang et al. 2009).

Balancing cell growth with division to maintain cellular homeostasis is a critical component of aging, whether cells are actively replicating or have entered a non‐proliferative state. Neurons are considered to be post‐mitotic and permanently arrested in the G_0_ phase of the cell cycle (Aranda‐Anzaldo and Dent 2017). However, neuronal cells that re‐enter the cell cycle will often fail to complete all cell cycle checkpoints and terminate in apoptosis or neurescence (neuronal cellular senescence) (Hudson et al. 2024; Jurk et al. 2012; Nandakumar et al. 2021; Riessland et al. 2019). In neurodegeneration, ectopic expression of cell cycle machinery and neuronal cell cycle re‐entry is commonly observed as an early precursor of NDDs (Herrup and Arendt 2002; Ruijtenberg and van den Heuvel 2016; Yang et al. 2006). Currently, there is ambiguity on how these post‐mitotic neurons re‐enter the cell cycle and where they arrest, as well as what pathways drive neuronal cell death versus neurescence. Yet, it is clear that aberrant activation of these cell‐cycle mechanisms in post‐mitotic neurons is a central process in neuronal aging and neurodegeneration.

Another point of intersection in aging and NDDs is the decline in function of nucleocytoplasmic transport (NCT), resulting in the intracellular redistribution of proteins and the accumulation of cytoplasmic aggregates such as TAR DNA‐Binding Protein‐43 (TDP‐43) (Kim and Taylor 2017). This nuclear loss of protein and toxic cytoplasmic gain of protein aggregates first implicated NCT, specifically nuclear import, in the pathogenesis of NDDs such as Alzheimer's disease (AD), amyotrophic lateral sclerosis (ALS), Parkinson's disease (PD), and Huntington's disease (HD) (Cruz and Cleveland 2016; Khalil et al. 2024). In the context of replicative aging, it has been shown that transport efficiency decreases in aged cells, consistent with a redistribution of proteins and dysfunctional nuclear pore complexes (Rempel et al. 2019). Recently, single nucleotide polymorphisms (R86S and R487G) in Nuclear Export Mediator Factor (NEMF) mouse models showed progressive motor neuron degeneration, characterized by hindlimb wasting and denervation of neuromuscular junctions (Martin et al. 2020). Utilizing this mouse model of neurodegeneration, we demonstrated that spinal motor neurons in these mice presented with cytoplasmic phospho‐TDP‐43 aggregates and mislocalized importin‐β (Plessis‐Belair et al. 2024), establishing this as a robust model for proteinopathy and neurodegeneration. Further investigations showed that the *Nemf* ^R86S^ mutant mice presented with NCT defects, specifically defective importin‐β nuclear import, as well as with transcriptional and phenotypical hallmarks of neurodegeneration (Plessis‐Belair et al. 2024). Furthermore, the Drosophila ortholog of NEMF, Caliban, has been previously described to mediate the G_1_/S transition through E2F1 regulation of the cell cycle (Song et al. 2018). Taken together, NCT defects, such as dysfunctional nuclear import, are a common pathological driver in neurodegeneration across various models of protein aggregation and neurodegeneration, including the NEMF mutant mouse model. The exact nature of how these NCT defects culminate in neurodegenerative phenotypes and the interplay between these defects and CCD has yet to be determined.

Here, we describe that an importin‐β nuclear import block in both mitotic neuronal cell lines and post‐mitotic primary neurons can dysregulate the cell cycle and result in cell cycle arrest. We further show that a G_1_/S cell cycle arrest is sufficient to cause the downregulation of stathmins, specifically *STMN2*. When this nuclear import block persists, a subpopulation of the cells will stochastically undergo apoptosis, while the surviving cells will transition into a senescence‐like state. This CCD is further described in our *Nemf* ^R86S^ mouse model through recapitulation of a G_1_/S cell‐cycle arrest. This cell‐cycle arrest is reiterated by aberrant expression of markers of CCD in both *Nemf* ^R86S^ primary neuronal cultures and spinal motor neurons. We provide evidence that defective importin‐β nuclear import drives CCD, which culminates in a cascade of transcriptional and homeostatic alterations. Our results implicate age‐ and gene‐driven dysfunction in NCT as a primary upstream mechanism driving neurodegeneration via CCD.

## 2 Methods

### 2.1 SK‐N‐MC Cell Culture

SK‐N‐MC cells (ATCC) were cultured according to the vendor's protocol. In brief, cells were maintained in ATCC‐formulated Eagle's Minimum Essential Medium (10% FBS, 1% PenStrep) at 37°C in 5% CO_2_. Reagents used are indicated in Table S1.

### 2.2 WT‐NEMF and R86S‐NEMF Mouse Embryonic Fibroblasts (MEFs) Derivation and Culture

MEFs were extracted and cultured as previously described (Plessis‐Belair et al. 2024). In brief, WT NEMF and R86S‐NEMF MEFs were maintained in Dulbecco's Modified Eagle's Medium (10% FBS, Glutamine 1X (Glutamax), 1% PenStrep) at 37°C, 5% CO_2_ and passaged every 2 days with Trypsin–EDTA (0.05%). Reagents used are indicated in Table S1.

### 2.3 Importazole Treatment Time Series

Cells were treated with 20 μM of importazole (Sigma) for 2, 12, 24, 48, 96 (4 days), and 168 (7 days) hours at 37°C, 5% CO_2_.

### 2.4 Cell Cycle Inhibitor Treatments

Cells were treated with Nocodazole (500 nM), Deferoxamine (10 μM), and L‐Mimosine (100 μM) for 48 h at 37°C 5% CO_2_.

### 2.5 Fluorescence Associated Cell Sorting (FACS) Cell Cycle Analysis

Cells were washed one time with PBS (1X) and trypsinized and resuspended in media. Suspended cells were centrifuged for 5 min at 500 × *g*. The supernatant was removed, and cells were resuspended in 1 mL of 70% Ethanol and incubated for at least 2 h with inversion. Cells were washed once with PBS (1X). Cells were centrifuged again for 5 min at 500 × *g*; the supernatant was removed, and cells were resuspended in 500 μL DAPI/PBS/triton X‐100 (0.1%). Cells were incubated for 30 min in the dark. FACS sorting was adjusted for excitation at 340 nm to 380 nm and detection of DAPI for G_1_/S/G_2_ discrimination.

### 2.6 Immunofluorescence Staining

As previously reported (Plessis‐Belair et al. 2024), cells were chemically fixed in 4% paraformaldehyde in PBS(1X). Each well was then rinsed with PBS(1X). The cells were then permeabilized with 0.1% Triton X‐100/PBS for 10 min at room temperature. The cells were then blocked for 1 h at room temperature with 5% NGS/PBS/0.1% Tween‐20. The cells were then incubated with the respective primary antibody in 5% NGS/0.1% Tween‐20/PBS overnight at 4°C. The primary antibodies used are listed in Table S1. The next day, the cells were rinsed 3 times for 5 min with 0.1% Tween‐20/PBS(1X). The cells are then incubated with the respective secondary antibodies in 5% NGS/0.1% Tween‐20/PBS for 2 h in the dark at room temperature. The secondary antibodies used are listed in Table S1. The cells were then rinsed 3 times for 5 min with 0.1% Tween‐20/PBS and stored temporarily with 200 μL of PBS(1X) in the dark at 4°C. Using sterile slides, 20–25 μL of Prolong glass with NucBlue (Thermo Fisher) was added to the slide. The liquid was then aspirated from the well and using an SE 5 tissue curved forceps, the coverslips were gently picked up and then placed with the cell layer (top) down on the slide. The slide was then cured in the dark at 4°C for 24 h. Confocal images were taken by an Olympus FV3000 Laser Scanning Confocal. Laser settings (laser strength, gain, and offset) and magnification were maintained across treatment groups. Post‐processing of images was performed by ImageJ and Cell Profiler as described below.

### 2.7 RNA Isolation

RNA was isolated from SK‐N‐MC or Primary Cortical Neurons utilizing the Purelink RNA Mini‐kit (Invitrogen). RNA concentrations were standardized through NanoDrop (Thermo Fisher) and snap frozen at −80°C.

### 2.8 RNA Seq of IPZ‐Treated SK‐N‐MC Neuronal Cells and *Nemf* ^R86S^ MEFs

Concentrated RNA was sent for bulk RNAseq to Azenta. In brief, sample quality control and determination of concentration were performed using TapeStation Analysis by Azenta, followed by library preparation and sequencing. Computational analysis included in their standard data analysis package was used for data interpretation. *Nemf* ^R86S^ MEFs RNA‐seq dataset was previously published (Plessis‐Belair et al. 2024).

### 2.9 Reconstruction and Inference of a Transcriptional Regulatory Networks

The reconstruction of transcriptional regulatory networks was performed as described in the TNI pipeline in the RTN package in R with the list of known human transcription factors (TFs) obtained from Fletcher et al. 2013. In brief, mutual information between TFs (1192 regulons) and all potential targets (14,022 targets) is computed by removing non‐significant associations through permutation analysis (nPermutations = 1000) with correction for multiple hypothesis testing (tni.permutation()). Unstable interactions are then removed through bootstrap analysis (tni.bootstrap()), creating a reference regulatory network (778,232 edges). Next, the ARACNe algorithm is applied which utilizes the direct processing inequality (DPI) theorem to enrich regulons by eliminating the weakest interactions between two TFs and a common target gene (tni.dpi.filter()). The resulting network formed is herein referred to as the transcriptional regulatory network (60,412 edges). From here, one can retrieve individual regulons and their weighted interactions with target genes (tni.get()).

### 2.10 Two‐Tailed Gene Set Enrichment Analysis

Two‐tailed gene set enrichment analyses are performed as described in the TNA pipeline in the RTN package in R. In brief, a transcriptional regulator analysis is performed to assess the overlap between each regulon and significantly differentially expressed genes in the dataset (tna.mra()). One‐tailed gene set enrichment analysis (GSEA1) assesses the interaction between a regulon and a ranked gene list generated from the differentially expressed genes. The regulons are then scored based on the association between the differentially expressed genes and the resulting response or phenotype. Two‐tailed gene set enrichment analysis (GSEA2) separates the differentially expressed genes into positive and negative targets based on Pearson's correlation between the regulon and the targets and then assesses the positive or negative association between the regulon and gene targets. To evaluate this phenotype, a differential enrichment score (dES) is calculated based on a stepwise evaluation of positive (ES*pos*) and negative(ES*neg*) gene enrichment scores. A positive dES represents an activated regulon, whereas a negative dES represents a repressed regulon activity.

### 2.11 Mitotracker and Lysotracker Visualization

For imaging of mitochondria and lysosomes, cells were plated on sterile 12 mm round glass coverslips and exposed to a 7‐day treatment with either DMSO or 20 μM IPZ. Following treatment, cells were washed with PBS 1X and then incubated for 15 min at 37°C in either 500 nM Mitotracker Red CMXRos in PBS 1X or 1 μM Lysotracker Deep Red in PBS 1X. Following treatment, cells were chemically fixed in 4% PFA and immunostained for other markers as described in *Immunofluorescence Staining*. Confocal images were taken by an Olympus FV3000 Laser Scanning Confocal. Laser settings (laser strength, gain, and offset) and magnification were maintained across treatment groups. Post‐processing of images was performed by ImageJ and Cell Profiler as described below.

### 2.12 miRNA Transfections

Transfections of scrambled (SCR) miRNA and miR22‐3p inhibitor were performed as previously described with minor adjustments (Russo et al. 2024). In brief, SK‐N‐MC cells were plated on 6‐well plates and treated for 4 days with 20 μM IPZ. At this time point, cells were transfected with SCR miRNA or miR22‐3p inhibitor (20 pmol). Media were changed 24 h after transfection, and RNA was isolated (*RNA Isolation*) 48 h after transfection.

### 2.13 Western Blotting

Western blotting was performed as previously described (Plessis‐Belair et al. 2024). In brief, cells were lysed with RIPA lysis buffer (Sigma) supplemented with protease inhibitor. Protein concentrations were standardized by Pierce BCA Protein Assay. 10 μg of lysate was prepared with 50 mM dTT (BioRad) and 4X Laemmli buffer (BioRad). The lysates were then loaded onto a Stain‐Free mini‐protean 10 well pre‐cast gel (BioRad) and mini‐protean tank (BioRad) with a Chameleon 800 MW ladder (Licor). The gels were run at 200 V for approximately 45 min in Tris/Glycine/SDS Running Buffer (BioRad). Proteins separated in gels were transferred to a PVDF membrane using a semi‐dry blotting method. Transfers were run for 90 min, with the current maintained between 80 mA–240 mA. The membrane is then incubated with TBS‐Based Odyssey blocking buffer for 1 h at room temperature. The membrane is then incubated in blocking buffer supplemented with 0.1% Tween‐20 and primary antibody overnight. The primary antibodies used are listed in Table S1. The membrane is then washed with TBS‐T (0.1% Tween‐20) 3 times for 5 min. The membrane is then incubated with blocking buffer (0.1% Tween‐20) and the respective secondary antibody at room temperature for 2 h. The secondary antibodies used are listed in Table S1. The membrane is then washed with TBS‐T 3 times for 5 min. The membrane is imaged with the Odyssey Scanner (Licor).

### 2.14 Antibody Array

Antibody array was prepared as described by Cell Cycle Phospho Array protocol by Full Moon Biosystems. In brief, proteins were extracted through RIPA Lysis as described above in *Western Blotting*. The resulting cell lysate was then purified through buffer exchange. The purified protein was then biotinylated using a Biotin/DMF solution (10 mg/mL). The antibody array slide is then blocked, rinsed with distilled water, and then the biotinylated protein is coupled onto the slide. The slide is briefly washed, and the proteins are detected through Cy3‐streptavidin. The slide is washed with wash buffer and then extensively rinsed with distilled water. The antibody array is then dried and scanned using a microarray scanner.

### 2.15 Mouse Strains, Husbandry, and Genotyping

All mouse husbandry and procedures were reviewed and approved by the Institutional Animal Care and Use Committee at Stony Brook University and were carried out according to the NIH Guide for Care and Use of Laboratory Animals. Tail tissue was lysed in proteinase K at 55°C overnight, and extracted DNA was used for genotyping. Genotyping for B6J‐*Nemf* ^R86S^ was performed via PCR using the following primers: forward primer specific to wild‐type allele: 5′‐AACATTTGAAGAGTCGGGGA‐3′, forward primer specific to mutant allele: 5′‐AACATTTGAAGAGTCGGGGT‐3′, reverse primer common for both alleles: 5′‐GCAGGTGGATGGTAGCAACG‐3′.

### 2.16 Primary Neuronal Cell Extraction and Culture

Primary neuronal cell extraction was performed as previously described (Plessis‐Belair et al. 2024). In brief, brains were quickly dissected from P0/P1 pups in 2 mL of Hibernate‐A/B27 (0.5 mM GlutaMAX, 1% PenStrep, 1% B27). Cortices were minced and digested in Papain Digestion Medium (100 units Papain in 2 mL Hibernate‐A). Slices were washed twice in 2 mL of Hibernate‐A/B27. Slices were then triturated 10 times with a siliconized 9‐in. Pasteur pipette with a tip fire polished to an opening of 0.7–0.9 μm diameter. Supernatants were then transferred to a new tube and the cells were pelleted by centrifugation at 80 × *g* for 5 min. Cells were then counted by hemocytometer. The remaining cell pellet was resuspended in an appropriate volume of Neurobasal‐A/B27 (1% B27, 1% PenStrep, 0.5 mM GlutaMax) and the cells were plated at 80% of plating volume. Neurobasal media was changed after 45 min of culture, and then was subsequently changed every 2 days.

### 2.17 Spinal Cord Immunostaining

Spinal cords were immunostained as previously described (Plessis‐Belair et al. 2024). In brief, spinal cords were surgically removed and chemically fixed in 4% PFA in PBS 1X. Spinal cords were placed in optimal cutting temperature (OCT) and stored in −80°C. Spinal cords were cryosectioned at −20°C into 30 μm sections and then stored in PBS 1X at 4°C. Sections were permeabilized in 0.3% TritonX‐100/PBS and blocked in 5% NGS/PBS/0.1% TritonX‐100. Sections were then incubated with the respective primary antibody in 5% NGS/PBS/0.1% TritonX‐100 overnight at 4°C. The primary antibodies used are listed in Table S1. The spinal cords were then incubated with the respective secondary antibodies in 5% NGS/PBS/0.1% TritonX‐100 for 2 h in the dark at room temperature. The secondary antibodies used are listed in Table S1. Confocal images were taken by an Olympus FV1000 Laser Scanning Confocal. Laser settings (laser strength, gain, and offset) and magnification were maintained across treatment groups. Post‐processing of images was performed by ImageJ and Cell Profiler as described below.

### 2.18 RT‐qPCR

RNA (200 ng) was reverse transcribed (Superscript IV Reverse Transcriptase (Thermo Fisher)) and the output volume of 20 μL was diluted in nuclease‐free water to 40 μL for a working concentration of 5 ng/μL. Real‐time PCR was performed using SYBR Green PCR Master Mix (Applied Biosystems) on a QuantStudio 3 System (Applied Biosystems) with reaction specificity confirmed by melt curve analysis. All comparisons (Control vs. Experimental) for each qPCR reaction were run on the same qPCR plate and were run in a triplicate. For qPCR primer sequence, see Table S2.

### 2.19 Image Analyses

Images were analyzed in bulk through Cell profiler. Z‐projections were taken from each image by maximum intensity and then separated by fluorophore. Nuclear/Cytoplasmic (N/C) Ratios were taken by comparing the area of the nucleus (DAPI) and the area of the cytoplasm (Phalloidin or brightfield). Nuclear and Cytoplasmic Intensities were standardized to area.

### 2.20 Statistical Analyses

Statistical tests were performed using GraphPad's Prism (v10) software. A threshold of *p* < 0.05 was considered significant. Significance was determined using the test indicated in the figure legend. Results are presented as means ± SD. Individual data points display individual cells or biological replicates as indicated.

## 3 Results

### 3.1 IPZ‐Treated Mitotic Neuronal Cell Lines Demonstrate Cell Cycle Dysregulation Consistent With the Downregulation of Stathmins

Previously, we described the effects of a transient nuclear import block using a small molecular antagonist of importin‐β, importazole (IPZ), on an SK‐N‐MC neuronal cell line (Plessis‐Belair et al. 2024). The observations of TDP‐43 proteinopathies and transcriptional dysregulation of *Stmn2* in this cell line implicated TDP‐43 regulatory dysfunction in these IPZ‐treated cells. It has been reported that human *STMN2* expression is directly regulated by TDP‐43 by binding to a cryptic exon that is lacking in mouse *Stmn2* (Baughn et al. 2023). However, IPZ treatment in mouse cell lines where TDP‐43 regulation of *Stmn2* is absent shows the same downregulation of *Stmn2*, highlighting that *STMN2* regulation is not TDP‐43‐exclusive, and that a parallel pathway exists downstream of nuclear import which can culminate in the observed transcriptional dysregulation.

**FIGURE 1:**
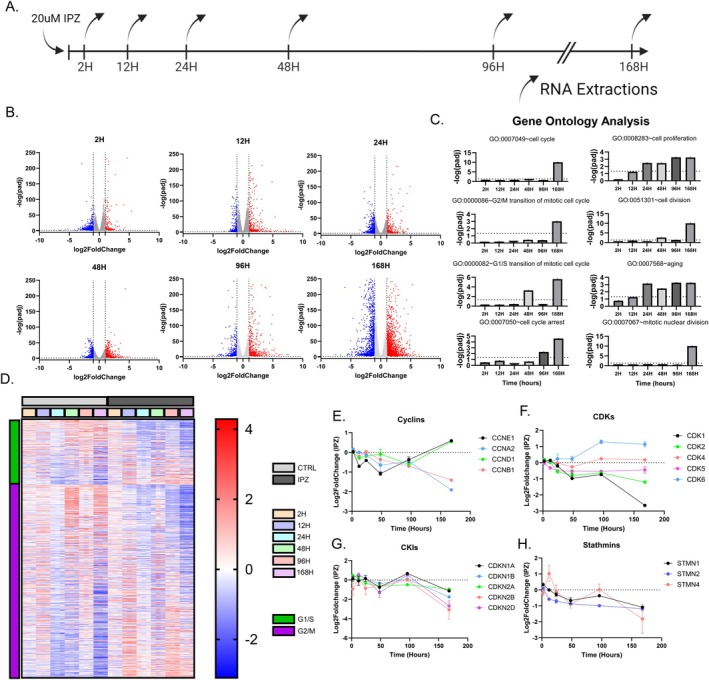
IPZ‐treated mitotic neuronal cell lines demonstrate cell cycle dysregulation consistent with the downregulation of stathmins. (A) Timeline of the experimental set up. Cells were treated for 7 days in parallel with RNA extractions occurring at 2, 12, 24, 48, 96, and 168 h. (B) Volcano plot of significant DEGs for each time point. Red data points indicated upregulated DEGs (log2FC > 1, *p*‐adj < 0.05) and blue data points indicate downregulated DEGs (log2FC < −1, *p*‐adj < 0.05). Gray data points indicate DEGs that do not meet the threshold (−1 < log2FC < 1|*p*‐adj > 0.05). (C) Gene Ontology Analysis of significant DEGs shown in (B) for each given timepoint (*p*‐adj < 0.05). (D) Z‐score heatmap of genes associated with G_1_/S and G_2_/M clusters. (E–H) Log2FoldChange Expression of Cyclins (E), CDKs (F), CKIs (G), Stathmins (H), over the 7‐day time course.

To elucidate the mechanisms involved in this parallel pathway, we isolated RNA from 2, 12, 24, 48, 96, and 168 h‐IPZ‐treated SK‐N‐MC cells (doubling time: 48 h) and performed bulk‐RNA sequencing (Figure 1A). With time, there is a clear increase in the number of significant differentially expressed genes (DEGs, *p*‐adj < 0.05, log2FC > 1|log2FC < −1), with 320 significant DEGs at 2H, 1442 DEGs at 48H, and 3972 DEGs at 168H. Gene Ontology (GO) analysis of significant DEGs at 48H highlights dysregulation of expressed genes in the regulation of cell proliferation (GO:0008283), cell division (GO:0051301), and aging (GO:0007568), specifically highlighting genes involved in the G_1_/S transition of the mitotic cell cycle (GO:0000082) (Figure 1B,C). The significance of DEGs enriched in the G_1_/S transition pathways disappears at 96H but returns at 168H (Figure 1C). However, the enrichment of DEGs observed in cell cycle arrest (GO:0007050) is observed at 96H and 168H (Figure 1C). Similarly, GO analysis reveals pathway enrichment in the G_2_/M transition of the cell cycle (GO:0000086), cell proliferation, cell division, aging, and mitotic nuclear division (GO:0007067) at 168H (Figure 1C).

Proliferating eukaryotic cells that undergo regular cell divisions can be separated into discrete cell cycle phases: G_1_, S, G_2_, and M (Israels and Israels 2000). The progression of cells from G_1_ to S and from G_2_ to M is regulated by restriction checkpoints (Blagosklonny and Pardee 2002). These checkpoints serve to detect errors in the cell cycle prior to progression into the next phase and will drive cell‐cycle arrest until the defect is repaired. To confirm whether these IPZ‐treated cells were arrested at G_1_/S or G_2_/M, we utilized fluorescence‐associated cell sorting (FACS) to isolate continuous DNA content profiles from IPZ‐treated cells at each time point (Figure S1A) (Jayat and Ratinaud 1993; Pozarowski and Darzynkiewicz 2004). Control cells showed slight fluctuations in the percentage of cells in G_1_, S, and G_2_ throughout the time course, but did not show any overt cell cycle arrest as indicated by shifts into each peak (Figure S1B). IPZ‐treated cells did not show any significant changes in the percentage of cells in G_1_, S, and G_2_ for the first 48 h (Figure S1B). However, cells then accumulated into G_1_ peaks at 96H which were maintained until 168H (Figure S1B). This increase in G_1_ with a consistent decrease in S and no significant changes in G_2_ suggests a G_1_/S cell‐cycle arrest initiated at around 96H (Figure S1B).

We then focused on genes associated with the G_1_/S and G_2_/M pathway and observed their differential expression over time (Figure 1D). Specifically, cyclins (Cyclin A, B, D, E) and cyclin‐dependent kinases (CDK 1, 2, 4, 6) will form complexes and function to phosphorylate proteins which are crucial for cell cycle progression (Giacinti and Giordano 2006). Here, we observed phasic expression of cyclins, with their expression becoming increasingly dysregulated with time until we observed a split in expression, with G_1_/S associated *CCNE1* and *CCND1* being upregulated and G_2_/M associated *CCNB1* and *CCNA1* being downregulated at 168H (Figure 1E; Figure S2A,B). Interestingly, CDK transcripts lack the phasic expression observed with cyclins, with *CDK6* showing an upregulation, and *CDK1* and *CDK2* showing a downregulation (Figure 1F). *CDK4* and *CDK5* show no change in expression (Figure 1F). The regulation of cell cycle checkpoints is further associated with CDK inhibitors (CKIs) which can be broken down into two main families, the Cip/Kip family (p21^Cip1^ (*CDKN1A*) and p27^Kip1^ (*CDKN1B*)) and the INK4 (Inhibitors of CDK4) gene locus (p16^INK4a^ (*CDKN2A*), p15^INK4b^ (*CDKN2B*), p18^INK4c^ (*CDKN2C*), p19^INK4d^ (*CDKN2D*)) (Fischer et al. 2003). Expression of CKIs similarly showed a more phasic cell‐cycle expression profile, with many CKIs (*CDKN1A*, *CDKN2A*, *CDKN2B*, and *CDKN2D*) showing a general downregulation in expression at 168H (Figure 1G; Figure S2C,D).

We sought to further investigate the dysregulation of cell cycle machinery by comparing the expression of long‐noncoding RNAs (lncRNAs) which have been implicated in both CCD and neurodegeneration (Hung et al. 2011; Kitagawa et al. 2013; Riva et al. 2016; Sun et al. 2015; Wan et al. 2017; Zhou et al. 2021). We analyzed significant DEGs of 5047 lncRNAs which were further categorized into anti‐sense, intronic, long interspersed noncoding (lincRNA), long‐noncoding non‐systematic (lnc Non‐systematic), microRNA, and small nucleolar RNA host genes (SNHG) (Figure S3A,B). Similar to the phasic expression of cell cycle components, lncRNAs showed phasic expression, with alternating downregulation/upregulation of significant DEGs (Figure S3A,C). Specifically, lncRNA *MEG3*, which has been implicated in neurodegeneration including AD, PD, and HD (Balusu et al. 2023; Chanda et al. 2018; Quan et al. 2020), showed an early upregulation at 48H with its expression returning to baseline by 168H (Figure S3D). In contrast, *MIR17HG* and *MIR22HG*, which have been implicated in AD and PD (Ning et al. 2022; Russo et al. 2024; Zhang et al. 2022), both show late upregulation at 168H with variable phasic expression throughout the time‐course (Figure S3D).

We further investigated the transcriptional dysregulation of simple repeats and transposable elements (TEs) which have been shown to be de‐repressed in neurodegeneration (Fondon et al. 2008; Li et al. 2012; Ravel‐Godreuil et al. 2021; Reilly et al. 2013; Wojciechowska et al. 2014). We analyzed significant DEGs of 15,295 simple repeats and TEs which were further categorized into DNA transposons, long interspersed nuclear elements (LINE), long terminal repeats (LTR), and short interspersed nuclear elements (SINE) (Figure S4A,B). Interestingly, we did not observe any phasic expression or dysregulation of simple repeats and TEs (Figure S4A,C). Rather, we saw a significant increase in dysregulated simple repeats and TEs at 168H, consistent with the large dysregulation observed with lncRNA (Figure S4C). Altogether, the ultimate dysregulation of lncRNA, simple repeats, and TEs suggests a general increase in heterochromatin relaxation consistent with aging and neurodegeneration. However, the phasic nature of dysregulation for lncRNA suggests a more direct association with the observed cell‐cycle dysregulation.

Stathmins have been shown to be transcriptionally regulated for microtubule stability and cell cycle progression (Polager and Ginsberg 2003; Polzin et al. 2004). The regulation of stathmins, specifically *STMN1* and *STMN2*, has been described to play an important role in motor neuron diseases and neurodegeneration (Baughn et al. 2023; Bellouze et al. 2016; Gagliardi et al. 2022; Klim et al. 2019; Krus et al. 2022; López‐Erauskin et al. 2024; San Juan et al. 2022). Our observed alterations in cell cycle machinery were further associated with the dysregulation of stathmins, where *STMN1*, *STMN2*, and *STMN4* all showed a general downregulation with IPZ‐treatment (Figure 1H; Figure S2E). Interestingly, *STMN4* showed phasic expression similar to other cell cycle components, whereas *STMN2* showed a general downward trend in expression and *STMN1* fell somewhere in between (Figure 1H; Figure S2E). Ultimately, inhibiting importin‐β function resulted in cell‐cycle dysregulation consistent with the cell‐cycle associated downregulation of stathmins.

### 3.2 IPZ Treatment Results in Time‐Dependent Cell‐Cycle Regulator Activity Dysfunction

**FIGURE 2:**
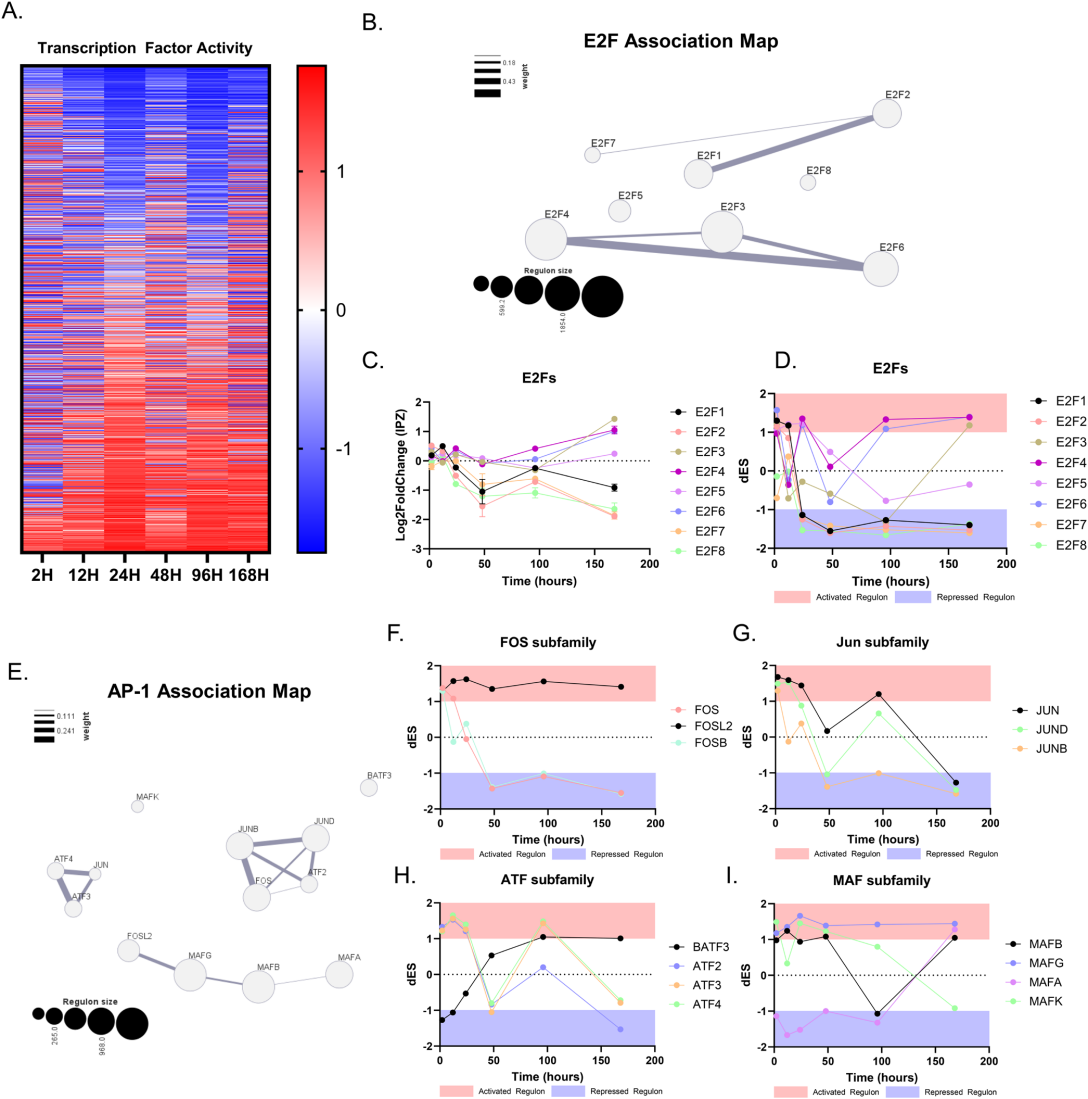
IPZ‐treated cell lines show time‐dependent cell‐cycle regulator activity dysregulation. (A) Differential Enrichment Score (dES) heatmap of IPZ‐treated SK‐N‐MC cells. The activity of 1192 transcription factors are inferred based on a reconstructed transcriptional network (RTN) from SK‐N‐MC gene expression profiles. (B) E2F Association Map inferred from the RTN displaying each regulon (E2Fs 1–8) and its size (represented by area), as well as overlapping associations in transcriptional activity with other regulons (measured by weighted line). (C) Log2FoldChange Expression of E2Fs 1–8 over the 7‐day time course. (D) dES transcriptional activity of E2Fs 1–8 over the 7‐day time course. (E) AP‐1 complex Association Map inferred from the RTN displaying each regulon family (FOS, JUN, ATF, and MAF). Size is represented by area as well as overlapping associations in transcriptional activity with other regulons (measured by weighted line). (F–I) dES transcriptional activity of FOS (F), JUN (G), ATF (H), and MAF (I) subfamilies over the 7‐day time course. Activated regulon activity in red shaded area (dES > 1) and repressed regulon activity in blue shaded area (dES < −1). Scatter plot bars are mean with standard deviation (C).

Given the critical role that the cell cycle plays in transcriptional regulation, we inferred and reconstructed a transcriptional network (RTN) of target genes and transcription factors (TFs) and subsequently performed two‐tailed gene set enrichment analysis to isolate the activity of transcriptional regulators over time (Fletcher et al. 2013). Applying the ARACNe algorithm utilizing the data processing inequality (DPI) theorem to remove redundant and unstable interactions, we constructed an RTN comprised of 1192 regulons consisting of 14,022 targets (see ‘Reconstruction and Inference of a Transcriptional Regulatory Networks’ in Methods). Regulon activity varied throughout the time course but remained balanced across samples throughout the RTN (Figure 2A).

From here, two‐tailed gene set enrichment analysis revealed enrichment of transcriptional regulators in the E2F family. The E2F family of TFs has been described as key regulators for cell cycle progression through the G_1_/S checkpoint (Cam and Dynlacht 2003; Zhu et al. 2004). We then reconstructed an association map and saw that E2F1 gene targets are linearly associated with E2F2 and E2F8, whereas E2F3 has a larger regulon size and greater association with E2F4 and E2F6 (Figure 2B). E2F5 and E2F8 are small regulons and do not show any preferential association in this RTN with other E2F TFs (Figure 2B). Observing the expression and activity of these E2F TFs revealed phasic expression profiles and activity of some TFs, but not all (Figure 2C,D). E2Fs 1, 2, 7, and 8 showed similar phasic expression patterns with a general downward trend in expression. Despite this phasic expression, these E2F TFs showed a significant repression in regulon activity as early as 24H which was maintained throughout the time course (Figure 2C). On the contrary, E2Fs 3, 4, 5, and 6 showed variable changes in expression correlated with an upregulation in expression and activity (Figure 2C,D). Altogether, we suggest the E2F family of TFs as a primary upstream regulator for the observed cell‐cycle associated transcriptional dysregulation.

AP‐1 TFs have been shown to regulate the cell cycle by activating and inhibiting the expression of key components of cell‐cycle machinery, including but not limited to *CCND1, CDKN1A, CDKN2B, CDKN2D*, and *TP53* (Shaulian and Karin 2001, 2002). Thus, we turned to the transcriptional activity of factors associated with the AP‐1 complex of transcriptional regulators. Here, we highlighted four subfamilies that can both hetero‐ and homo‐dimerize to regulate transcriptional activity: Jun, FOS, MAF, and ATF (Bohmann et al. 1987; Chinenov and Kerppola 2001; Fujiwara et al. 1993; Kataoka et al. 1995; Liebermann et al. 1998; Shaulian and Karin 2001). The construction of an AP‐1 association map demonstrates that ATF4, ATF3, and JUN have high association and relatively small regulon sizes, whereas JUNB, JUND, FOS, and ATF2 show strong association of regulatory targets and slightly larger regulon sizes (Figure 2E). FOSL2, MAFG, MAFB, and MAFA are observed to be linearly associated, each with relatively large regulon sizes (Figure 2E). The activity of the FOS subfamily can be separated into FOS/FOSB, which shows similar early and sustained repressed activity, whereas FOSL2 shows a maintained active regulon (Figure 2F). The Jun subfamily shows a more phasic activity pattern, with all three members JUN, JUND, and JUNB showing late repression of regulon activity (Figure 2G). The ATF subfamily consisting of ATFs 2, 3, and 4 showed phasic activity similar to the Jun subfamily, with the exception of BATF3, which becomes increasingly activated with time (Figure 2H). The MAF subfamily showed highly variable activity, with MAFA, B, and G showing a late activated regulon activity and MAFK showing a downward trend to repressed activity (Figure 2I).

Taken together, the time‐dependent activity profiles provide insight on cell‐cycle related activity changes versus general repression or activation of TFs as a result of the nuclear import inhibition, further highlighting nuclear import‐mediated cell‐cycle dysfunction.

### 3.3 Cell‐Cycle Dysregulation Is Associated With Senescence‐Like Features Independent of CKI Expression

The presence of CCD in the absence of CKI expression (*CDKN1A*, *CDKN2A/B*, *CDKN2D*, Figure 1G) even at our latest time points raises confounding questions on diverging pathways between apoptosis and senescence. Therefore, we aimed to explore whether surviving cells at 168 h demonstrated canonical hallmarks of a senescence‐like phenotype, including senescence‐associated secretory phenotype (SASP), reduced lamin expression and an increase in nuclear size, mitochondrial and lysosomal dysfunction, and DNA damage (Martínez‐Cué and Rueda 2020; Russo and Riessland 2022).

**FIGURE 3:**
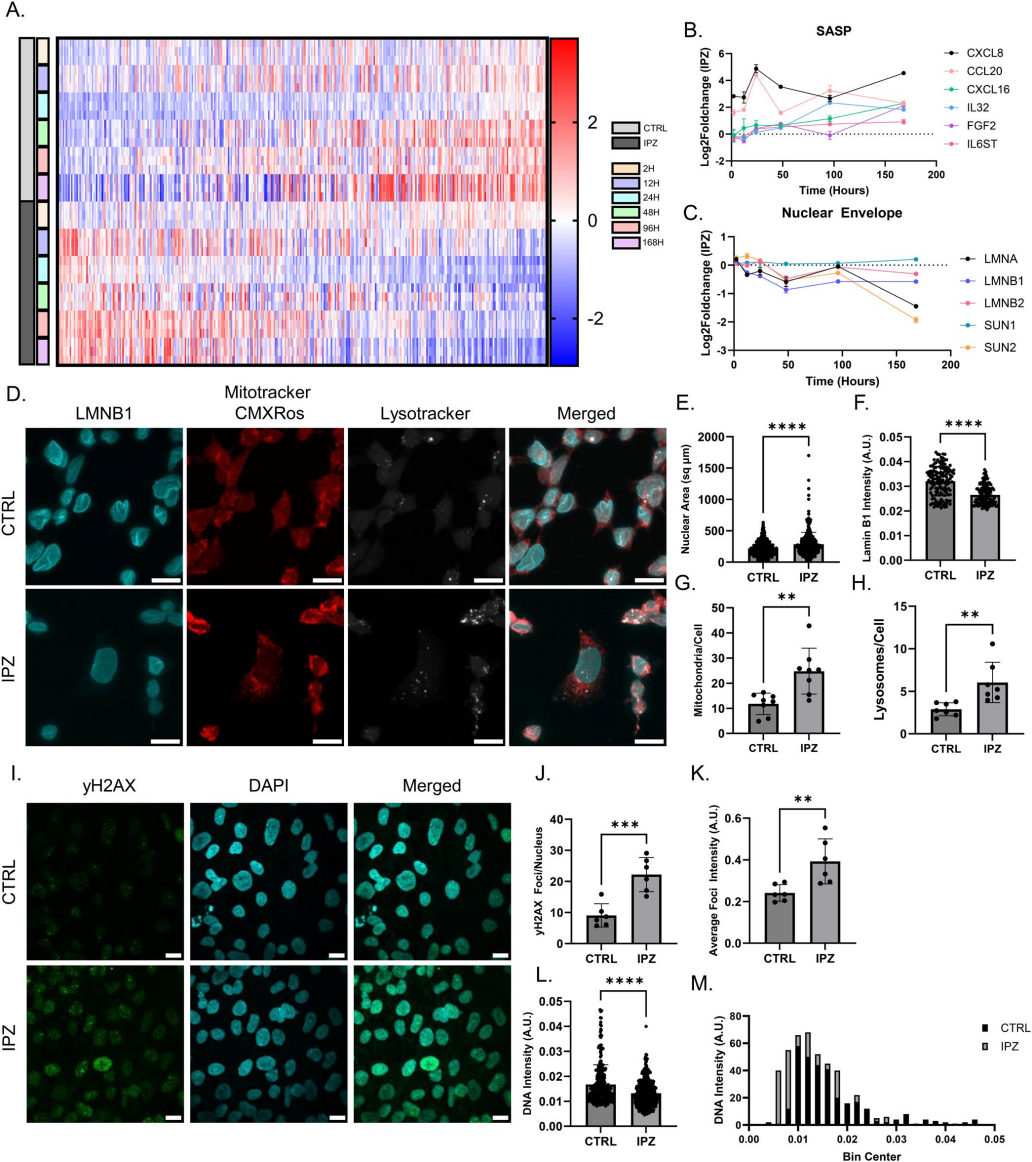
Cell‐cycle dysregulation is associated with senescence‐like features independent of CKI expression. (A) Z‐score heatmap of genes associated with senescence and SASP. (B, C) Log2FoldChange Expression of SASP (B) and Nuclear Envelope (C) DEGs over the time course of 7‐days. (D) Immunostaining of lamin B1 (blue) with Mitotracker CMXRos (red) and Lysotracker stains (white) in CTRL and IPZ‐treated SK‐N‐MC cells. (E) Nuclear area (μm^2^) of CTRL and IPZ‐treated SK‐N‐MC cells (*n* = 304–379 cells). (F) lamin B1 fluorescence intensity from (D) (*n* = 128–134). (G, H) Quantification of average mitochondria and lysosomes per cell isolated from Mitotracker CMXRos and Lysotracker stains from (D) (*n* = 8 trials). (I) Immunostaining of γH2AX (Ser 139, green) and DAPI (blue) in CTRL and IPZ‐treated SK‐N‐MC cells. (J) Quantification of average γH2AX foci per nucleus isolated from (I). (K) Average γH2AX fluorescence intensity per foci from (I) (*n* = 6). (L) DNA intensity from DAPI staining from isolated from CTRL and IPZ‐treated SK‐N‐MC cells from (I) (*n* = 303–421). All data was analyzed by unpaired two‐tailed *t*‐test. (**p < 0.01, ***p < 0.001, ****p < 0.0001). (M) Frequency distribution histogram of DNA intensity from (L) for CTRL and IPZ‐treated SK‐N‐MC cells. Scale bars are 10 μm.

Utilizing a previously established set of genes identified in senescence and the SASP (SenMayo) (Saul et al. 2022), we examined the expression of these genes over time following nuclear import inhibition and observed a significant dysregulation of genes associated with the SASP (Figure 3A). Specifically, *CXCL8* and *CCL20* upregulation occurred as early as 2 h post‐treatment. Whereas *CXCL8* expression varies but ultimately increases to 168H, CCL20 shows a slight comparative decrease in expression at the same time point (Figure 3B; Figure S2F,G). In comparison, *CXCL16*, *IL32*, *FGF2*, and *IL6ST* showed a time‐dependent response correlated with a gradual increase in expression (Figure 3B). Interestingly, the most upregulated SASP factors lacked a phasic expression profile, suggesting that their upregulation was a cumulative response (Figure 3B). Examinations into nuclear envelope transcriptional regulation revealed a slight yet significant downregulation of lamins, specifically *LMNA* at 168H. *LMNB1* shows an initial drop in expression up to 48H, with its comparative expression increasing slightly yet still remaining significantly downregulated (Figure 3C; Figure S2H). In comparison, *LMNB2* showed less dysregulation but similar expression patterns (Figure 3C). Interestingly, the dysregulation of *LMNA* was strongly correlated with the phasic dysregulation of LINC complex component *SUN2*, but not *SUN1*, suggesting cell‐cycle‐associated regulation of these gene transcripts which become increasingly more dysregulated with time (Figure 3C) (Haque et al. 2010). We then followed up on the dysregulation of lamins through immunostaining of the senescent biomarker lamin B1, whose quantification showed a significant increase in nuclear size consistent with a decrease in lamin B1 intensity (Figure 3D–F). Qualitatively, the lamin B1 immunostaining displayed a subpopulation of cells with large, flat nuclei, with very few folds in the nuclear envelope, which is consistent with a senescence‐like phenotype (Figure 3D) (González‐Gualda et al. 2021; Neurohr et al. 2019).

Next, we investigated mitochondrial and lysosomal dysfunction through immunostaining and examination into associated DEGs. Mitotracker CMXRos staining showed an increase in the number of mitochondria per cell, suggesting an accumulation of mitochondria with time in these IPZ‐treated cells (Figure 3D,G). Consistent with these observations, we observed a broad phasic dysregulation of the top upregulated and downregulated DEGs associated with mitochondrial function, suggesting that the expression of mitochondrial genes is strongly correlated with the cell‐cycle dysfunction in our model (Figure S5A–C). Investigations into lysosomal dysfunction through immunostaining with lysotracker similarly showed an increased accumulation of lysosomes per cell, suggesting a lack of lysosomal turnover in treated cells (Figure 3D,H). Further investigations into the transcriptional regulation of lysosomal DEGs showed time‐dependent lysosomal dysregulation, with clusters of upregulated genes at 48H, as well as 96H and 168H (Figure S6A). Of these lysosomal genes, we observed an early upregulation of *LAMP3* as early as 12H, which is maintained throughout the time course (Figure S6B). However, other lysosomal genes showed a more gradual upregulation in expression, with many of them showing the highest differential expression at 48H, which is then either maintained (*CTNS*, *STS*, *HPS1*, *TPP1*) or observed to return to baseline (*CTSF*) (Figure S6C). Lastly, we examined the presence of DNA damage in these IPZ‐treated cells at 168H through immunostaining of the molecular marker γH2AX (Ser 139). We observed a significant increase in both the number of γH2AX foci per nuclei and the average γH2AX foci intensity with IPZ treatment (Figure 3I–K). We then looked at the distribution of DNA intensity through DAPI staining in these cells to look for DNA replication events through 2n (Bin Center 0.005–0.02 ± 0.005) and 4n (Bin Center 0.025–0.040 ± 0.005) DNA content populations (Figure 3L,M). Consistent with our observations through FACS (Figure S1), control cells displayed a left shift (increased 2n or G_1_) in DNA content, with a handful of cells displaying a DNA replication event (i.e., 4n DNA content). In contrast, IPZ‐treated cells show a wider left skew distribution, with little to no cells in the 4n DNA content range (Figure 3L,M). Due to the biological relevance of *MIR22HG* in both the context of CCD and cellular senescence and our observations of increased *MIR22HG* expression at 96H (4 days) and 168H (7 days) (Figure S3) (Russo et al. 2024), we targeted a derivative of this transcript, miR22‐3p, with artificial miRNA inhibitors (miR22‐3p inhibitor) and scrambled control following a 4 day IPZ treatment (Figure S7A). We observed no significant changes in cell cycle regulatory proteins (*CCND1, CCNE1, CDKN1A, CDKN2A*) (Figure S7B–F), but we did observe a restoration of senescence‐associated *CXCL8* (downregulated) and *LMNB1* (upregulated) relative to the IPZ condition (Figure S7G,H). Overall, this suggests that the function of *MIR22HG* is downstream of the CCD, yet upstream of senescence‐like features, highlighting its role as a potential regulatory factor of senescence phenotypes.

Overall, we demonstrate that cell‐cycle dysregulation and arrest contribute to the induction of an immune response in the form of SASP, the downregulation of lamin and nuclear envelope regulators, mitochondrial and lysosomal dysfunction, and ultimately, DNA damage, all of which are classical hallmarks of a senescence‐like phenotype.

### 3.4 *Nemf* ^R86S^ MEFs Demonstrate G_1_ /S Cell‐Cycle Arrest and *Stmn2* Downregulation

**FIGURE 4:**
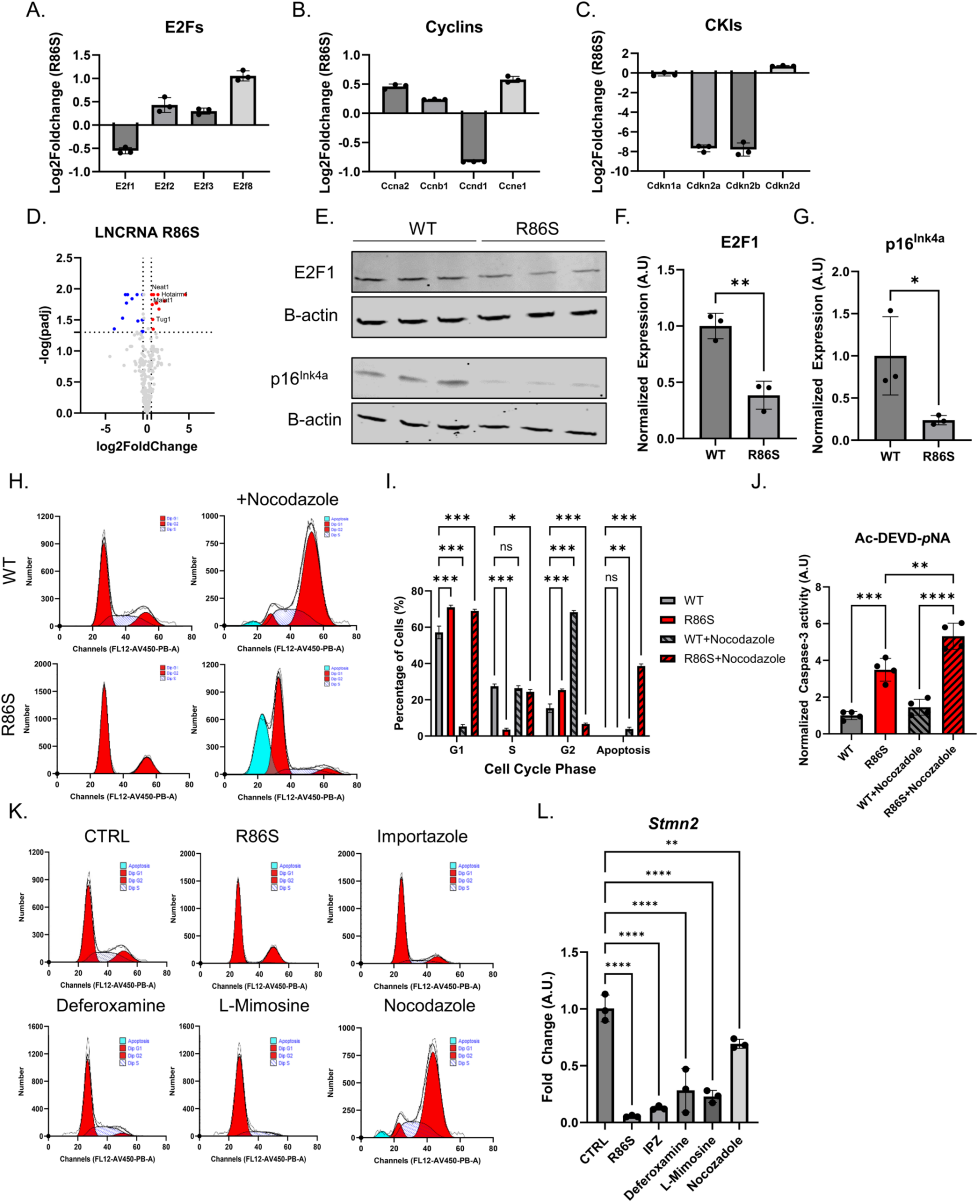
*Nemf* ^R86S^ MEFs demonstrate a G_1_/S cell‐cycle arrest and *Stmn2* downregulation. (A–C) Log2FoldChange Expression from *Nemf* ^R86S^ MEFs for E2Fs (A), Cyclins (B), and CKIs (C). (D) Volcano plot analysis of long non‐coding RNA DEGs. Red data points indicated upregulated DEGs (log2FC > 0.5, *p*‐adj < 0.05) and blue data points indicate downregulated DEGs (log2FC < −0.5, *p*‐adj < 0.05). Gray data points indicate DEGs that do not meet the threshold (−0.5 < log2FC < 0.5|*p*‐adj > 0.05). (E) Western blot analysis of E2F1 and p16^INK4A^ with respective β‐Actin loading control. (F, G) Quantification of protein expression from western blot analysis in (E). Data analyzed by unpaired two‐tailed *t*‐test (*n* = 3). (H) FACS of DNA content from DAPI staining of WT and *Nemf* ^R86S^ MEFs with and without Nocodazole treatment. (I) Quantification of the percentage of cells from (H) separated into G_1_, S, G_2_, and apoptosis peaks based on DNA content. Data from (H) analyzed by two‐way ANOVA with Šídák's multiple comparisons test (*n* = 3). (J) Colorimetric Caspase‐3 activity assay measuring AC‐DEVD‐pNA cleavage for WT and *Nemf* ^R86S^ MEFs with and without Nocodazole treatment normalized to WT (*n* = 4). (K) FACS of DNA content from DAPI staining of Control (WT) and R86S MEFs, as well as WT MEFs treated with Importazole, Deferoxamine, L‐Mimosine, and Nocodazole. (L) Quantitative PCR analysis of *Stmn2* RNA isolated from Control (WT) and R86S MEFs, as well as WT MEFs treated with Importazole, Deferoxamine, L‐Mimosine, and Nocodazole (*n* = 3). Data from (J, L) analyzed by ordinary one‐way ANOVA with Tukey's multiple comparison test. (ns *p* > 0.05, *p < 0.05, **p < 0.01, ***p < 0.001, ****p < 0.0001).

We previously described that *Nemf* ^R86S^ mouse embryonic fibroblasts (MEFs) showed an importin‐β specific nuclear import defect consistent with our observations in IPZ‐treated human neuronal cell lines (Plessis‐Belair et al. 2024). This prompted us to investigate whether the R86S mutation was capable of inducing CCD similar to the observed dysregulation we describe in IPZ‐treated cells. To test this hypothesis, we investigated the differential expression of E2Fs, cyclins, and CKIs in this R86S MEF model. We observed a slight yet significant downregulation of *E2f1*, as well as an increase in *E2f8*, with insignificant changes in *E2f2/3* transcripts (Figure 4A). We further observed differential expression of cyclins, with *Ccnd1* being downregulated and *Ccne1* being upregulated (Figure 4B). Interestingly, we observed no dysregulation of *Cdkn1a* and *Cdkn2d*, but a large downregulation of the *INK4* locus *Cdkn2a/b* (Figure 4C). This dysregulation of cell‐cycle machinery was consistent with the observed dysregulation of key lncRNAs such as *Neat1*, *Malat1*, *Tug1*, and *Hotairm1*, which have been observed to be dysregulated in neurodegeneration and our IPZ‐treated human cells (Figure 4D). Further investigation into protein expression of E2F1 and p16^Ink4a^ shows significant downregulation (Figure 4E–G), consistent with the observed transcriptional dysregulation (Figure 4A). We then utilized a semi‐quantitative antibody array for proteins expressed in the cell cycle and observed aberrant activation of many key proteins such as variants of phospho‐TP53, cyclin E1, and Cdc2, as well as the downregulation of Smad3 and Chk2 (Figure S8).

Next, we utilized FACS of DAPI‐stained WT and *Nemf* ^R86S^ MEFs, which shows the distribution of DNA content (Figure 4H). WT MEFs showed a G_1_/S/G_2_ distribution of 55/28/17 (%), highlighting that most cells exist in G_1_, with some cells undergoing mitosis by cycling through S/G_2_ phase (Figure 4H). The *Nemf* ^R86S^ MEFs showed a 71/4/25 (%) distribution, suggesting that most cells are arrested in G_1_ and G_2_, with few cells undergoing DNA replication in S phase (Figure 4I). To then further confirm if the cells were arrested in G_1_ or G_2_, we induced a G_2_ arrest through nocodazole treatment, which is used to disrupt microtubules, arresting cells in G_2_/M, as seen through treatment of WT MEFs (Figure 4H,I). Interestingly, *Nemf* ^R86S^ MEFs failed to arrest in G_2_ and remained in G_1_, suggesting that a majority of the cells are arrested in G_1_/S and cannot pass the restriction checkpoint (Figure 4H,I). Concurrently, nocodazole treatment induced an increase in apoptosis in both WT and *Nemf* ^R86S^ MEFs, as indicated by fractionated DNA as observed in FACS, with the *Nemf* ^R86S^ MEFs showing a significant percentage of cells in this apoptosis peak (Figure 4H,I). This significant increase in apoptosis is in line with an increase in caspase‐3 activity as measured through AC‐DEVD‐pNA cleavage in the *Nemf* ^R86S^ MEFs (Figure 4J).

Our previous characterization of *Nemf* ^R86S^ MEFs showed that the importin‐β specific nuclear import defect was consistent with the dysregulation of key genes in neurodegeneration. Among these dysregulated transcripts, we had previously described *Stmn2* to be significantly downregulated (Plessis‐Belair et al. 2024). Thus, we wanted to determine if bypassing the nuclear import block and inducing cell‐cycle arrest can similarly result in the dysregulation of *Stmn2*. Treatment of MEFs with IPZ resulted in a G_1_/S arrest as shown through FACS DNA intensity distributions and similarly resulted in a significant downregulation of *Stmn2* (Figure 4K,L). Therefore, we utilized Deferoxamine and L‐Mimosine, which have been previously shown to arrest cells in the G_1_/S phase (Fukuchi et al. 1997; Park et al. 2012). Deferoxamine is a known iron chelator resulting in iron deprivation, resulting in late G_1_ arrest, while L‐Mimosine has been shown to inhibit DNA replication, resulting in an early S phase arrest (Fukuchi et al. 1997; Park et al. 2012). Transient treatment of WT MEFs with both of these pharmacological agents indeed induced G_1_/S cell cycle arrest as well as a significant downregulation in *Stmn2* transcript levels (Figure 4K,L). Interestingly, cell cycle arrest in G_2_/M utilizing Nocodazole resulted in a slight reduction in *Stmn2* transcript levels, but not to the degree observed with G_1_/S arrest (Figure 4L).

Overall, these findings suggest that the *Nemf* ^R86S^ mutation results in CCD consistent with a G_1_/S cell cycle arrest and that cell cycle arrest in G_1_/S is sufficient to induce *Stmn2* transcript downregulation in a mouse mitotic cell line. These findings highlight an important key feature that CCD may be a consequence of a nuclear import block, yet upstream of the transcriptional dysregulation observed in neurodegeneration.

### 3.5 Mutant *Nemf* ^R86S^ and IPZ‐Treated Primary Neuronal Cultures Demonstrate Time‐Dependent Transcriptional Dysregulation Consistent With Cell Cycle Dysregulation

**FIGURE 5:**
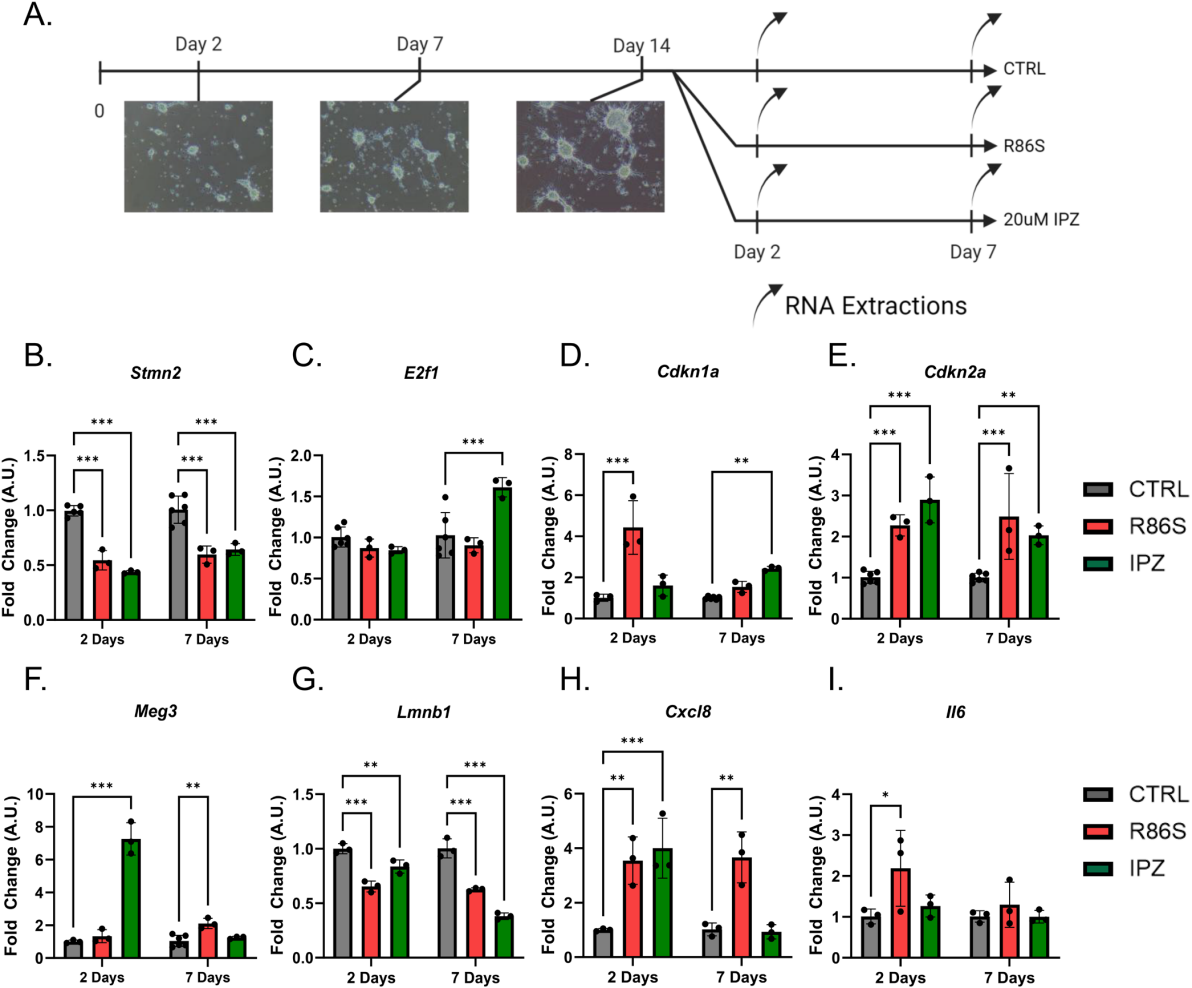
Mutant *Nemf* ^R86S^ and IPZ‐treated primary neuronal cultures demonstrate time‐dependent transcriptional dysregulation consistent with cell‐cycle dysregulation. (A) Timeline of the experimental set up. Primary cortical neurons were isolated at P0 from the same litter and cultured for 2 weeks. At the 2‐week time point, CTRL cells (WT mice) were treated with IPZ (20 μM) for either 2 or 7 days. CTRL and *Nemf* ^R86S^ (R86S) primary cultures were maintained in parallel with IPZ‐treated cells, with RNA extractions at 2 and 7 days. (B–I) Quantitative PCR of CTRL, R86S, and IPZ‐treated primary cortical neurons of *Stmn2* (B), *E2f1* (C), *Cdkn1a* (D), *Cdkn2a* (E), *Meg3* (F), *Lmnb1* (G), *Cxcl8* (H), *Il6* (I) at 2 and 7 days. Data from (B–I) analyzed by two‐way ANOVA with Šídák's multiple comparisons test. (*n* = 3) (*p < 0.05, **p < 0.01, ***p < 0.001).

To establish these observed defects in a post‐mitotic neuronal cell model, we isolated primary cortical neurons from WT and *Nemf* ^R86S^ mice from the same litter at P0 and cultured the isolated cells for 2 weeks. At this 2‐week time point, we treated WT primary neurons with 20 μM IPZ for 2 days (2 weeks, 2 days) and 7 days (3 weeks) and simultaneously maintained the respective control (CTRL) and *Nemf* ^R86S^ neurons in culture for each respective time point (Figure 5A). At a given time point, we extracted RNA and performed RT‐qPCR for target genes (*Stmn2*, *E2f1*, *Cdkn1a*, *Cdkn2a*, *Meg3*, *Lmnb1*, *Cxcl8*, *Il6*) previously observed to be dysregulated in IPZ‐treated SK‐N‐MC cells (Figure 2) and *Nemf* ^R86S^ MEFs (Figure 4). Consistent with our previous study, we observed a significant downregulation of *Stmn2* transcripts in both the R86S and IPZ‐treated primary neurons at both 2 days and 7 days (Figure 5B). Interestingly, we observed no significant dysregulation of *E2f1* at 2 days, but an upregulation of *E2f1* in IPZ‐treated primary neurons at 7 days (Figure 5C).

Next, we investigated the expression of CKI genes *Cdkn1a* and *Cdkn2a*, which have been shown to be upregulated in neurons following aberrant cell‐cycle re‐entry and neurescence (Hudson et al. 2024). The expression of *Cdkn1a* and *Cdkn2a* is highly variable in neurodegeneration, with the specific upregulation of *Cdkn1a* in neurons being associated with aging and PD (Hudson et al. 2024; Jurk et al. 2012; Riessland et al. 2019) and the upregulation of *Cdkn2a* being more closely associated with AD (Rödel et al. 1996; Vazquez‐Villaseñor et al. 2020). We found a significant upregulation of Cdkn1a at 2 days, but not at 7 days in *Nemf* ^*R86S*^(Figure 5D). Concurrently, *Cdkn1a* levels were not significantly different in the IPZ‐treated primary neurons at 2 days but were significantly increased at 7 days (Figure 5D). *Cdkn2a* expression in R86S and IPZ‐treated primary neurons was found to be significantly upregulated at both 2 days and 7 days, with *Cdkn2a* levels slightly decreasing in IPZ‐treated but remaining upregulated relative to control at 7 days (Figure 5E). Lastly, we observed a drastic upregulation in lncRNA *Meg3* for the IPZ‐treated primary neurons at 2 days, which was no longer found to be statistically different at 7 days (Figure 5F). Conversely, *Meg3* showed no significant differences at 2 days but was later found to be significantly upregulated in the R86S primary neurons at 7 days (Figure 5F).

To further validate a senescence‐like phenotype, we looked at the expression of the senescence‐associated nuclear envelope gene *Lmnb1* and SASP factors *Cxcl8* and *Il*6 in our primary neuronal cultures. *Lmnb1* was significantly downregulated at 2 and 7 days in the R86S primary neurons, consistent with a senescence‐like phenotype (Figure 5G). Expression of *Lmnb1* in IPZ‐treated primary neurons was slightly downregulated at 2 days and significantly downregulated at 7 days, suggesting a consistent decrease in *Lmnb1* expression with time (Figure 5G). We further observed a significant increase in the SASP marker *Cxcl8* expression at 2 days and 7 days in the R86S primary neurons (Figure 5H). However, the expression of *Cxcl8* increased at 2 days in IPZ‐treated primary neurons but returned to control levels at 7 days (Figure 5H). Expression of *Il6* in the R86S was significantly upregulated with high variation across replicates at 2 days, but insignificant at 7 days (Figure 5I). *Il6* expression was insignificant at both time points in IPZ‐treated primary neurons (Figure 5I). Altogether, the transcriptional dysregulation observed suggests neuronal cell‐cycle re‐entry through the aberrant expression of CKIs *Cdkn1a* and *Cdkn2a*. This neuronal‐associated cell‐cycle dysregulation is consistent with our observed downregulation of *Stmn2* and time‐specific expression of lncRNA *Meg3*. Furthermore, the downregulation of senescence‐associated *Lmnb1* and upregulation of SASP factors in these post‐mitotic neurons suggests senescence‐like features following chronic nuclear import defects.

### 3.6 Differential Expression and Localization of CKIs in *Nemf* ^R86S^ and IPZ‐Treated Post‐Mitotic Neurons

**FIGURE 6:**
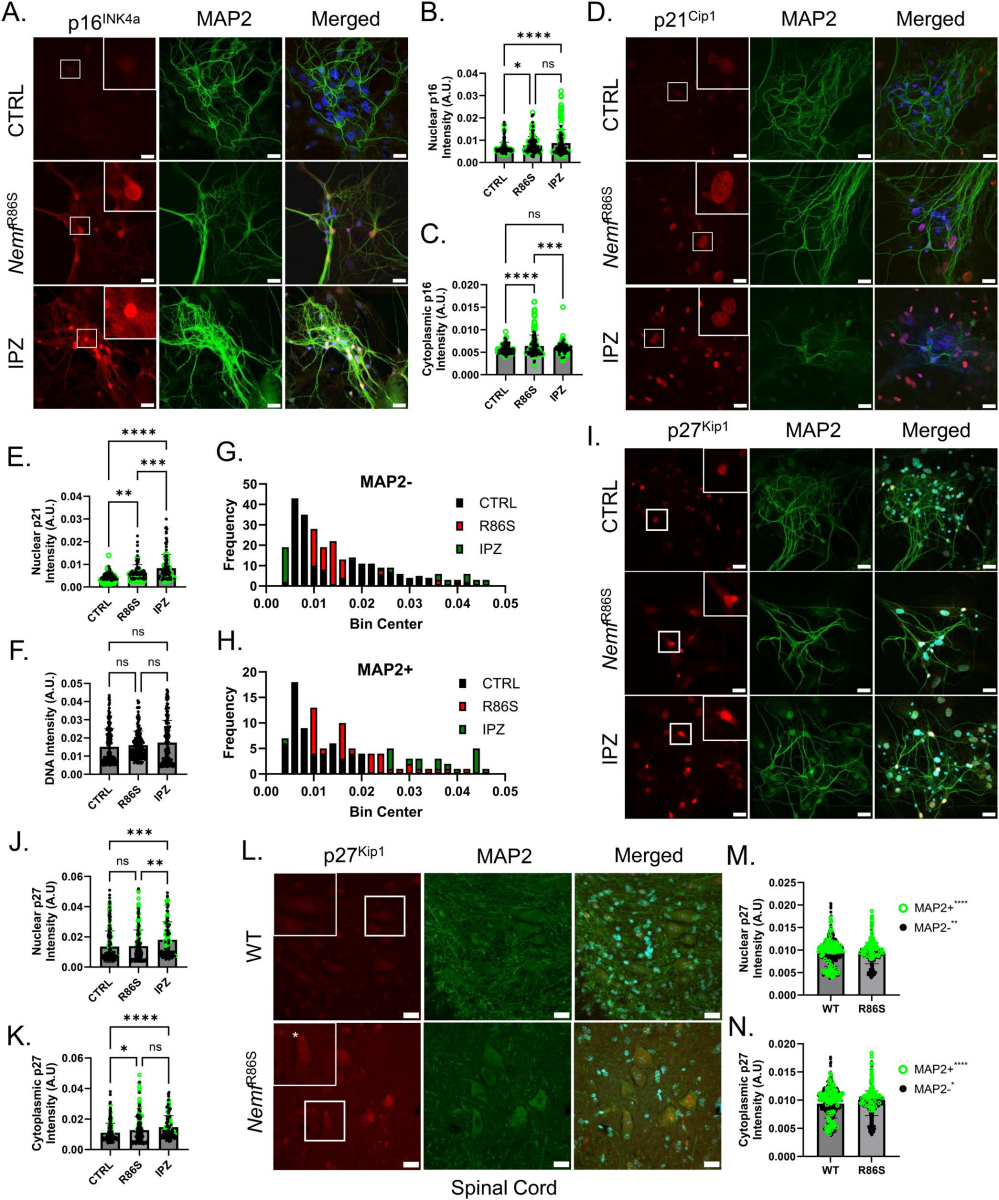
Differential expression and localization of CKIs in *Nemf* ^R86S^ and IPZ‐treated post‐mitotic neurons (A) Immunostaining of p16 (A, red), MAP2 (green), and DAPI (blue) in CTRL, *Nemf* ^R86S^ (R86S), and IPZ‐treated primary cortical neuronal cultures. (B, C) Nuclear (B) and cytoplasmic (C) p16 intensity from (A) isolated from neuronal (MAP2+, green open circle) and non‐neuronal (MAP2‐, black closed circle) cells (*n* = 166–185 cells). (D) Immunostaining of p21 (red), MAP2 (green), and DAPI (blue) in CTRL, *Nemf* ^R86S^ (R86S), and IPZ‐treated primary cortical neuronal cultures. (E) Nuclear p21 intensity from (D) isolated from neuronal (MAP2+, green open circle) and non‐neuronal (MAP2−, black closed circle) cells (*n* = 104–118 cells). (F) DNA intensity from DAPI staining isolated from neuronal (MAP2+, green open circle) and non‐neuronal (MAP2−, black closed circle) cells (*n* = 225–244 cells). Data from (C–F) analyzed by ordinary one‐way ANOVA with Tukey's multiple comparison test. (ns *p* > 0.05, *p < 0.05, **p < 0.01, ***p < 0.001, ****p < 0.0001). (G, H) Frequency distribution histograms from (F) separated into MAP2− cells and MAP2+ cells. (I) Immunostaining of p27 (red), MAP2 (green), and DAPI (blue) in CTRL, *Nemf* ^R86S^ (R86S), and IPZ‐treated primary cortical neuronal cultures. (J, K) Nuclear (J) and cytoplasmic (K) p27 intensity from (I) isolated from neuronal (MAP2+, green open circle) and non‐neuronal (MAP2−, black closed circle) cells (*n* = 120–266 cells). (L) Immunostaining of p27 (red), MAP2 (green), and DAPI (blue) in the ventral horn of WT and *Nemf* ^R86S^ spinal cord sections. (M, N) Nuclear (M) and cytoplasmic (N) p27 intensity from (L) isolated from neuronal (MAP2+, green open circle) and non‐neuronal (MAP2−, black closed circle) cells (*n* = 111–120 MAP2+ cells, 506–536 MAP2− cells). Scale bars are 20 μm.

Based on our observations of transcriptional dysregulation of cell‐cycle components and senescence‐associated biomarkers, we immunostained CTRL, R86S, and IPZ‐treated primary neurons at the 7‐day (3‐week total) time point for CKIs p16^INK4a^ (*Cdkn2a*) and p21^Cip1^ (*Cdkn1a*). The expression of p16^Ink4a^ and p21^Cip1^ under CTRL conditions was relatively low, with faint and diffuse signals in the nuclei of both neuronal and non‐neuronal cells (Figure 6A–E). The expression of p16^Ink4a^ in R86S primary cells was found to be uniquely expressed in MAP2+ neuronal cells (Figure 6A). The localization of p16^Ink4a^ was diffuse in the cytoplasm of these R86S primary neurons, with relatively low expression in the nucleus (Figure 6A–C). In contrast, expression of p16^Ink4a^ in IPZ‐treated primary neurons was found to be exclusive to the nucleus of MAP2+ cells, with relatively low expression in the cytoplasm (Figure 6A–C). Therefore, the discrepancy between the localization of p16 between the IPZ‐treated and R86S primary neurons is not a result of defective importin‐β nuclear import and suggests activity‐dependent localization (Mendaza et al. 2020; Vazquez‐Villaseñor et al. 2020). The expression of p21^Cip1^ in R86S and IPZ‐treated primary cells was found to be exclusively nuclear, with expression in both neuronal and non‐neuronal cells (Figure 6D,E). However, cells expressing the highest levels of p21^Cip1^ were predominantly non‐neuronal cells, contrary to the observed p16^Ink4a^ expression (Figure 6E).

Expression of p16^Ink4a^ and p21^Cip1^ in both R86S and IPZ‐treated primary cells highlights cell‐cycle re‐entry in these neuronal cells consistent with a senescence‐like phenotype. We then investigated whether there was also a correlation with an increase in total DNA levels as measured by DNA intensity from DAPI staining (Sigl‐Glöckner and Brecht 2017). We observed an overall increase in total DNA staining in the R86S (0.01628 A.U.) and IPZ‐treated (0.01843 A.U.) groups relative to control (0.013847 A.U.) which consisted of both MAP2+ and MAP2− cells (Figure 6F). However, DNA intensity distributions for control conditions showed only non‐neuronal cells with above‐average intensities, suggesting that MAP2+ neuronal cells are not actively cycling (Figure 6F). We then calculated the frequency of cells that fell within a DNA intensity bin range of ± 0.002 (Figure 6G,H). We found that CTRL primary cells correlated with a left‐skewed distribution, consistent with the predominant population falling in the G_0_/G_1_ range (Figure 6G). Isolating only MAP2+ shows that the majority of these neuronal cells were found within this G_0_/G_1_ range (Figure 6H). In contrast, R86S cells showed a slight rightward shift in DNA intensity in both MAP2+ and MAP2− populations, suggesting that neuronal cells might be undergoing a DNA replication event or might be further arrested in a late G_1_/S phase (Figure 6G,H). IPZ‐treated MAP2+ and MAP2− populations showed a wide range of DNA intensities with two distinct populations consistent with a DNA replication event, suggesting that many of these cells, including neuronal cells, might have undergone polyploidization (Nandakumar et al. 2021) (Figure 6G,H).

Whereas p21^Cip1^ and p16^Ink4a^ have a shared history through their well characterized role in mediating cell cycle progression and promoting cellular senescence, p27^Kip1^, a member of the Cip/Kip family, has emerged as a regulator of both cell cycle progression, autophagy, cell cytoskeletal dynamics, and apoptosis (Kawauchi et al. 2006; Liang et al. 2007; Polyak et al. 1994; Toyoshima and Hunter 1994). The functions of p27^Kip1^ have been described to be dependent on its subcellular localization, with its ability to regulate the cell cycle being associated with nuclear p27^Kip1^, whereas cytoplasmic p27^Kip1^ enhances cell survival through mediation of autophagy and cytoskeletal dynamics (White et al. 2018). Furthermore, there is strong evidence that the age‐related changes in both the expression and localization of p27^Kip1^ play an integral role in promoting quiescence, senescence, and apoptosis in both healthy and aging cells (Liang et al. 2007; White et al. 2018). Contrary to the expression of p16^Ink4a^ and p21^Cip1^, examinations into the expression and localization of p27^Kip1^ in primary cortical neuronal cultures revealed a diffuse nuclear localization in both neuronal and non‐neuronal cells in control conditions (Figure 6I–K). In R86S samples, we observed significant cytoplasmic localization, particularly in MAP2+ cells, but observed no general increase in nuclear expression across cell types (Figure 6I–K). Interestingly, we saw both an increase in the nuclear expression and cytoplasmic localization of p27^Kip1^ across conditions, suggesting both an upregulation in protein expression as well as a mis‐localization (Figure 6I–K).

We then turned to spinal motor neurons in *Nemf* ^R86S^ mice, which were previously described as the source of pathology in this model of neurodegeneration (Martin et al. 2020; Plessis‐Belair et al. 2024). Interestingly, these spinal motor neurons did not express p16^Ink4a^ or p21^Cip1^ but were observed to express p27^Kip1^. Immunostaining revealed subpopulations of both neuronal (MAP2+) and non‐neuronal (MAP2−) cells which either positively or negatively stained for p27^Kip1^ (Figure 6L–N) In contrast, most spinal motor neurons in *Nemf* ^R86S^ mice expressed p27^Kip1^ with increased expression in both the nucleus and the cytoplasm (Figure 6L–N).

Considering the role of CKIs in mediating the cell cycle as well as the role of CKI expression in neurodegeneration, expression of p16^Ink4a^, p21^Cip1^, and p27^Kip1^ in R86S and IPZ‐treated neuronal and non‐neuronal cells as well as an increase in DNA content in these neurons supports our hypothesis that defective nuclear import mechanisms culminate in cell‐cycle re‐entry in post‐mitotic cells.

## 4 Discussion

Nucleocytoplasmic transport defects are increasingly being recognized as a universal feature of physiological aging and NDDs (Hutten and Dormann 2020). Specifically, dysfunctional nuclear import has been observed downstream of both sporadic and familial models of neurodegeneration such as ALS, AD, and HD (Cunningham et al. 2020; Dubey et al. 2024; Eftekharzadeh et al. 2018; Gasset‐Rosa et al. 2017; Hayes et al. 2020; Zhang et al. 2015). Furthermore, post‐mitotic neurons reveal age‐dependent deficiencies in nucleocytoplasmic transport (D'Angelo et al. 2009; Mertens et al. 2015). Therefore, this unifying pathological hallmark of nuclear import defects in NDDs and the resulting CCD offers an important avenue for pathway‐specific therapeutic interventions.

When investigating dysregulation of the cell cycle as a potential pathway downstream of nuclear import defects, one must consider the implications of the fluctuating gene expression associated with the baseline mechanisms involved in the cell cycle. In particular, it is important to consider the downstream pathways involved in preparing a cell for mitosis, including but not limited to microtubule reorganization (Hasezawa et al. 1991; Sato and Toda 2010), DNA synthesis and replication (Sclafani and Holzen 2007; Stillman 1996), and nuclear envelope dynamics (Blow and Laskey 1988; Foisner 2003). These crucial pathways are often a requirement for the proper cycling of mitotic cells, and any perturbations within these pathways would result in cell‐cycle arrest, apoptosis, or cancer (Blumenfeld et al. 2017; Chow et al. 2012; Macheret and Halazonetis 2015; Sallee and Feldman 2021). However, the occurrence of these cell‐cycle‐associated pathways in post‐mitotic neurons, such as the reorganization of microtubules or the nuclear envelope, may have detrimental effects on neuronal health. Therefore, neuronal cell‐cycle re‐entry events may be unwanted byproducts of integral mechanisms in cellular homeostasis. The evidence provided here for cell‐cycle re‐entry in both *Nemf* ^R86S^ and IPZ‐treated primary neurons highlights dysregulation of these crucial cell‐cycle associated pathways through the fluctuation of gene expression, downstream of nuclear import defects, culminating in a neurodegenerative phenotype.

Specifically, we focus on the expression of *Stmn2* following cell‐cycle arrest at the G_1_/S transition. The activation of the CDK‐RB‐E2F pathway observed in G_1_, which drives E2F‐regulated transcriptional activation, is required for progression through the G_1_/S checkpoint (Johnson et al. 1994; Nevins 2001; Zhu et al. 2004). It has been previously shown that activation of E2F TFs can upregulate stathmin transcripts through interactions with the stathmin promoter, and thus inhibition of E2F transcriptional activity either through loss of E2F or through inhibition of the CDK‐RB‐E2F pathway by CKIs can result in decreased stathmin expression (Iancu‐Rubin and Atweh 2005; Polzin et al. 2004). In particular, repression of AP‐1 component JUN has been implicated in the reduction of stathmin expression (Kinoshita et al. 2003). Here, we describe the repression of E2F and AP‐1 activity downstream of CKI transcriptional downregulation, suggesting that nuclear import defects may initially propagate CCD, independent of CKI inhibition of the CDK‐RB‐E2F axis. In the case of post‐mitotic neuronal cells, we observe aberrant upregulation of CKIs p16^Ink4a^ and p21^Cip1^ associated with cell cycle re‐entry, highlighting differences in CKI expression between mitotic and post‐mitotic cells. Interestingly, investigations into p27^Kip1^ suggest that the subcellular localization of these CKIs may play an integral role in their function. In particular, p27^Kip1^ has been previously described to inhibit stathmin directly through its cytoplasmic localization, or indirectly through the CDK‐RB‐E2F axis (Baldassarre et al. 2005; Polzin et al. 2004). Altogether, we suggest that defective nuclear import‐induced CCD is a direct upstream pathway resulting in the downregulation of stathmins, specifically *Stmn2*, in NDDs.

Additionally, we describe that these IPZ‐treated mitotic cells demonstrate senescence‐like features through the observed expression of the SASP, reduced lamin expression, mitochondrial and lysosomal dysfunction, and DNA damage. The presence of senescence‐like hallmarks further suggests that perturbations in cell‐cycle regulation alone can drive cellular senescence phenotypes. Our discrepant observations of CKI expression between mitotic and post‐mitotic cells further elucidate cell‐type specific differences in the expression of senescence‐like hallmarks, which are all downstream of cell‐cycle dysregulation. Therefore, we suggest a novel and reproducible model to induce features of cellular senescence in vitro by impairing nuclear import and consequently inducing CCD. Overall, our findings demonstrate that there is a direct impact on the cell cycle through importin‐β‐mediated nuclear import inhibition, culminating in CCD in mitotic cells as well as inducing cell‐cycle re‐entry in post‐mitotic primary cortical neurons. We further showed the phasic and variable expression of key genes and biomarkers observed to be dysregulated in neurodegeneration, suggesting that cell‐cycle re‐entry has a strong influence on transcriptional regulation. To this end, we predicted and described repressed transcriptional activity of specific transcriptional regulators in the cell cycle, such as E2Fs and AP‐1. This dysregulation in transcriptional activity was further associated with the downregulation of *STMN2*. This pathological cell‐cycle re‐entry was also recapitulated in the mutant *Nemf* ^R86S^ post‐mitotic primary cortical neurons which displayed a significant upregulation of CKIs *Cdkn1a* (p21^Cip1^) and *Cdkn2a* (p16^Ink4a^) both through quantitative PCR and immunostaining, with p16^Ink4a^ expression being neuronal specific and p21^Cip1^ being broadly expressed across cell types. Additionally, we showed that p27^Kip1^ protein expression is upregulated in both the nucleus and the cytoplasm in our R86S and IPZ‐treated primary neurons, as well as in R86S spinal motor neurons. Lastly, we described in these R86S and IPZ‐treated primary neurons a significant downregulation of *STMN2*, and upregulation of the SASP factors *Cxcl8 and Il6* as well as downregulation of *Lmnb1*. Altogether, the data suggest that cell‐cycle dysregulation is downstream of importin‐β nuclear import defects, culminating in cell‐cycle re‐entry in post‐mitotic neurons and models of neurodegeneration. Importantly, our findings have therapeutic implications as they suggest targeting nuclear import receptors or enhancing their performance could help to reduce these cell cycle re‐entry events and concurrently, reduce the toxic burdens faced by these post‐mitotic neurons.

## Author Contributions

Conceptualization: Roger Sher, Jonathan Plessis‐Belair, Markus Riessland, Taylor Russo. Formal analysis: Roger Sher, Jonathan Plessis‐Belair. Funding acquisition: Roger Sher. Investigation: Roger Sher, Jonathan Plessis‐Belair, Taylor Russo. Methodology: Roger Sher, Jonathan Plessis‐Belair, Taylor Russo. Project administration: Roger Sher. Resources: Roger Sher. Validation: Roger Sher, Jonathan Plessis‐Belair. Visualization: Roger Sher, Jonathan Plessis‐Belair. Writing – original draft: Roger Sher, Jonathan Plessis‐Belair. Writing – review and editing: Roger Sher, Jonathan Plessis‐Belair, Markus Riessland, Taylor Russo.

## Disclosure

We permit the right to Wiley and Aging Cell to license and reproduce the above information. We required no permissions for any data or figures produced in this manuscript.

## Conflicts of Interest

The authors declare no conflicts of interest.

## Supporting information


Data S1.

## Data Availability

The data that support the findings of this study are openly available OSF at https://osf.io/6vwpd/, reference number 10.17605/OSF.IO/6VWPD.
