# Supplementary material for: Nuclear Import Defects Drive Cell Cycle Dysregulation in Neurodegeneration
Source: Aging Cell. 2025 May 16;24(8):e70091. doi: 10.1111/acel.70091 (PMC12341789; doi:10.1111/acel.70091)
Supplement: Supplementary file 1 — Data S1. [file ACEL-24-e70091-s001.zip › acel70091-sup-0001-DataS1.docx]

**Supplementary Figures**

**Fig S1: IPZ-treated mitotic neuronal cell lines display a G_1_/S cell-cycle arrest through DNA distribution.** A) FACS of DNA content from DAPI staining of Control and IPZ-treated SK-N-MC cells for 2, 12, 24, 48, 96, and 168 hours. B) Percentage of cells from (A) found in the G_1,_ S, and G_2_ cell cycle phase across each time point.

**Fig S2: qPCR validation of dysregulated RNA-seq transcripts at 2 days and 7 days post IPZ-treatment** A-H) Quantitative PCR of CTRL and IPZ-treated SK-N-MC cells of *CCND1* (A), *CCNE1* (B)*, CDKN1A* (C)*, CDKN2A* (D)*, STMN2* (E)*, CCL20* (F)*, CXCL8* (G)*, LMNB1* (H) at 2 and 7 days. Data from (A-H) analyzed by two-way ANOVA with Šídák's multiple comparisons test. (n=4) (* p<0.05, ** p<0.01, *** p<0.001).

**Fig S3: Dysregulation of lncRNA DEGs follows cell-cycle associated phasic expression.** A) Volcano plot analysis showing only significant lncRNA DEGs at 2, 12, 24, 48, 96, and 168 hours separated into Antisense, Intronic, lincRNA, lnc Non-Systematic, MicroRNA, and SNHG (padj<0.05, log2FC<-1|log2FC>1). B) Distribution (%) of significant non-Coding DEGS from (A). C) Quantification of upregulated and downregulated significant non-coding DEGs over the 7-day time-course. D) Log2FoldChange Expression of lncRNA *MEG3*, *MIR17HG*, and *MIR22HG* over the 7-day time course.

**Fig S4: Dysregulation of Simple Repeats and Transposable Elements occurs late following IPZ treatment.** A) Volcano plot analysis showing only significant Repeat and TE DEGs at 2, 12, 24, 48, 96, and 168 hours separated into DNA Transposons (DNA), LINE, LTR, Sime Repeats, and SINE (padj<0.05, log2FC<-1|log2FC>1). B) Distribution (%) of significant Repeat and TE DEGS from (A). C) Quantification of upregulated and downregulated significant Repeat and TE DEGs over the 7-day time-course.

**Fig S5. Cell-cycle dependent dysregulation of mitochondrial-associated DEGs.** A) Z-score heatmap of Mitochondrial DEGs. B-C) Log2FoldChange Expression of Upregulated (B) and Downregulated (C) Mitochondrial DEGs over the time course of 7-days.

**Fig S6: Upregulation of lysosomal-associated DEGs with IPZ treatment.** A) Z-score heatmap of Lysosomal DEGs. B-C) Log2FoldChange Expression of LAMPs (B) and other Lysosomal (C) DEGs over the time course of 7-days.

**Fig S7: Suppression of lncRNA *MIR22HG* diminishes senescence-like features** A) Schematic of experimental workflow. SK-N-MC cells are treated with 20µM IPZ for 4 days then treated with either scrambled or miR22-3p inhibitor. RNA was extracted 48 hours later. B-H) Quantitative PCR of *MIR22HG* (B), *CCND1* (B), *CCNE1* (C)*, CDKN1A* (E)*, CDKN2A* (F)*, CXCL8* (G)*, LMNB1* (H) from SK-N-MC cells treated for 4 days with IPZ and then treated with scrambled and miR22-3p. Data from (B-H) analyzed by unpaired two-tailed t-tests (n=3) (* p<0.05, ** p<0.01, *** p<0.001).

**Fig S8: Phosphorylated Cell-Cycle Protein Expression Antibody Array.** Heatmap of the expression of each detected protein normalized to a beta-actin loading control.

| **Cell Culture** | **Company** | **Cat #** |
| --- | --- | --- |
| Dulbecoo’s Modified Eagle’s Medium | Corning | 10-017-CV |
| Eagle’s Minimum Essential Medium | ATCC | 30-2003 |
| FBS 1X | Gibco | 26140079 |
| Penstrep 100X | Gibco | 15140122 |
| Glutamax 100X | Gibco | 35050061 |
| Non-essential Amino-Acids 100X | Gibco | 11140050 |
| Trypsin-EDTA (0.05%) | Gibco | 25300062 |
| Hibernate-A | Gibco | A1247501 |
| Neurobasal-A | Gibco | 10888022 |
| B27 | Gibco | 17504044 |
| **Antibody** | **Company** | **Cat #** |
| MAP2 | AVES | 4H5 |
| p16INK4a | Invitrogen | PA5-20379 |
| p21 | Invitrogen | MA5-31479 |
| Lamin B1 | Abcam | Ab16048 |
| yH2AX (Ser139) | Cell Signaling | 20E3 |
| Alexa Fluor 594 g@ms IgG2a | Invitrogen | A21135 |
| Alexa Fluor 488 g@Rb Ig | Invitrogen | A11034 |
| Alexa Fluor 488 g@Chk IgY | Invitrogen | A11039 |
| Alexa Fluor 594 g@Chk IgY | Invitrogen | A11042 |
| Alexa Fluor 594 g@ms IgG1 | Invitrogen | A21125 |
| Alexa Fluor 594 g@Rb Ig | Invitrogen | A11037 |
| Alexa Fluor 594 g@ms IgG2b | Invitrogen | A21141 |
| Alexa Fluor 405 g@Rb Ig | Invitrogen | A31556 |
| IRDye 800CW Goat anti-Mouse IgG1 | Licor | 926-32350 |
| IRDye 800CW Goat anti-Rabbit IgG | Licor | 926-32211 |

**Table S1: List of Cell Culture Reagents and Antibodies.**

| **Primer** | **Forward** | **Reverse** |
| --- | --- | --- |
| *Gapdh (mouse)* | GGCAAATTCAACGGCACAGT | GGGTCTCGCTCCTGGAAGAT |
| *Stmn2 (mouse)* | TGTCACTGATCTGCTCCTGC | TGGGAGATGGTGGCTTCAAG |
| *Gapdh (human)* | GTCTCCTCTGACTTCAACAGCG | ACCACCCTGTTGCTGTAGCCAA |
| *Actb (mouse)* | CATTGCTGACAGGATGCAGAAGG | TGCTGGAAGGTGGACAGTGAGG |
| *Cdkn1a (mouse)* | TCGCTGTCTTGCACTCTGGTGT | CCAATCTGCGCTTGGAGTGATAG |
| *Cdkn2a (mouse)* | TGTTGAGGCTAGAGAGGATCTTG | CGAATCTGCACCGTAGTTGAGC |
| *Cxcl8 (mouse)* | CCTTTCCACCCCAAATTTAT | AAACTTCTCCACAACCCTCTG |
| *Il6 (mouse)* | TACCACTTCACAAGTCGGAGGC | CTGCAAGTGCATCATCGTTGTTC |
| *E2f1 (mouse)* | GGATCTGGAGACTGACCATCAG | GGTTTCATAGCGTGACTTCTCCC |

**Table S2: List of Primers.**
