# Supplementary material for: Nuclear Import Defects Drive Cell Cycle Dysregulation in Neurodegeneration
Source: Aging Cell. 2025 May 16;24(8):e70091. doi: 10.1111/acel.70091 (PMC12341789; doi:10.1111/acel.70091)
Supplement: Supplementary file 1 — Data S1. [file ACEL-24-e70091-s001.zip › Nuclear_Import_Defects_Drives_Cell_Cycle_Dysregulation_supplemental figures only.pdf]

Nuclear Import Defects Drive Cell Cycle Dysregulation

Jonathan Plessis-Belair[1,2], Taylor Russo[1,2], Markus Riessland[1,2], and Roger Sher[1,2]

Figure S1

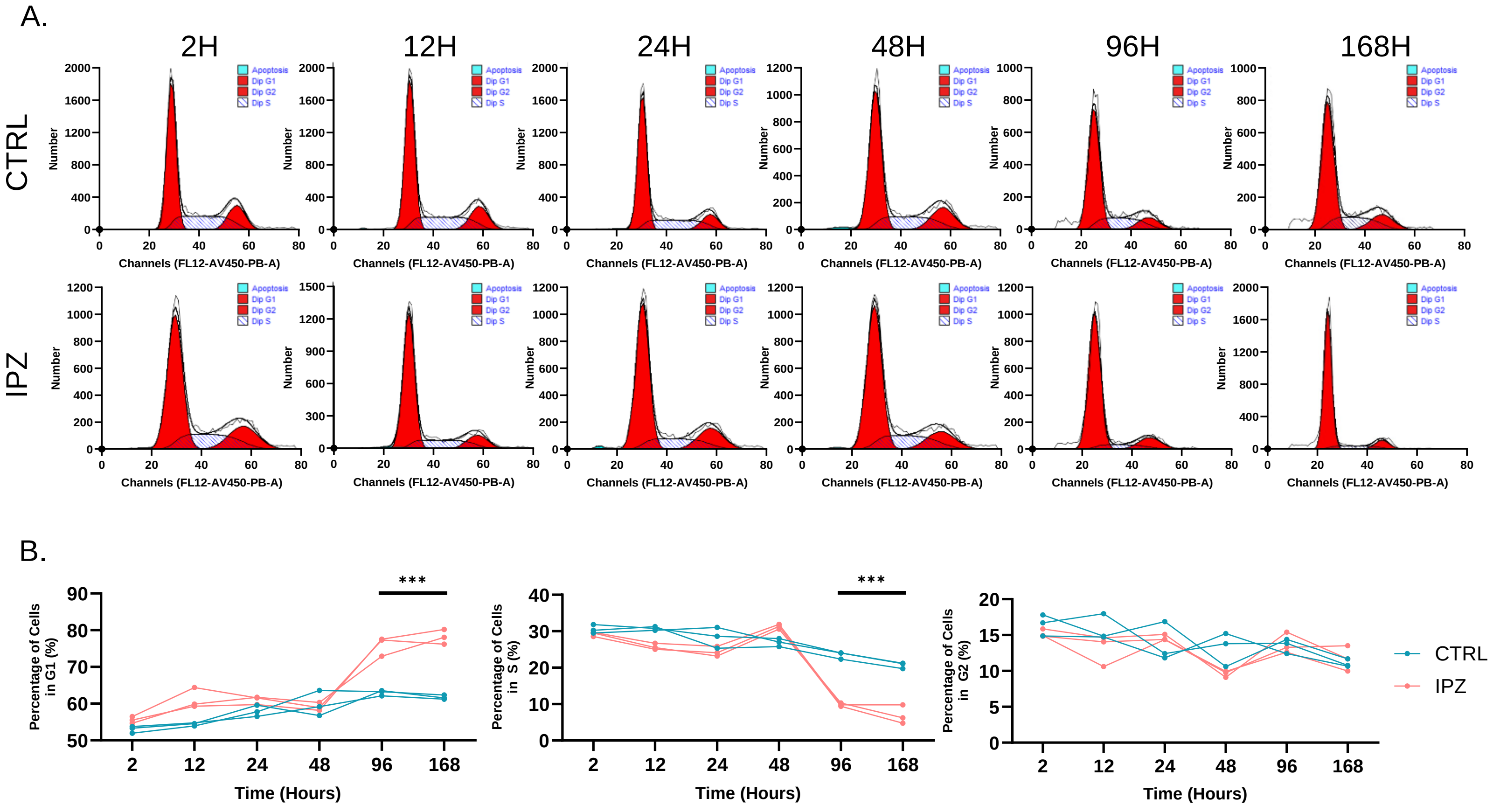

A.
2H
12H
24H
48H
96H
168H
CTRL
IPZ
Apoptosis
Dip G1
Dip G2
Dip S
Number
Channels (FL12-AV450-PB-A)
B.
Percentage of Cells in G1 (%)
Percentage of Cells in S (%)
Percentage of Cells in G2 (%)
Time (Hours)
***
CTRL
IPZ

Figure S2

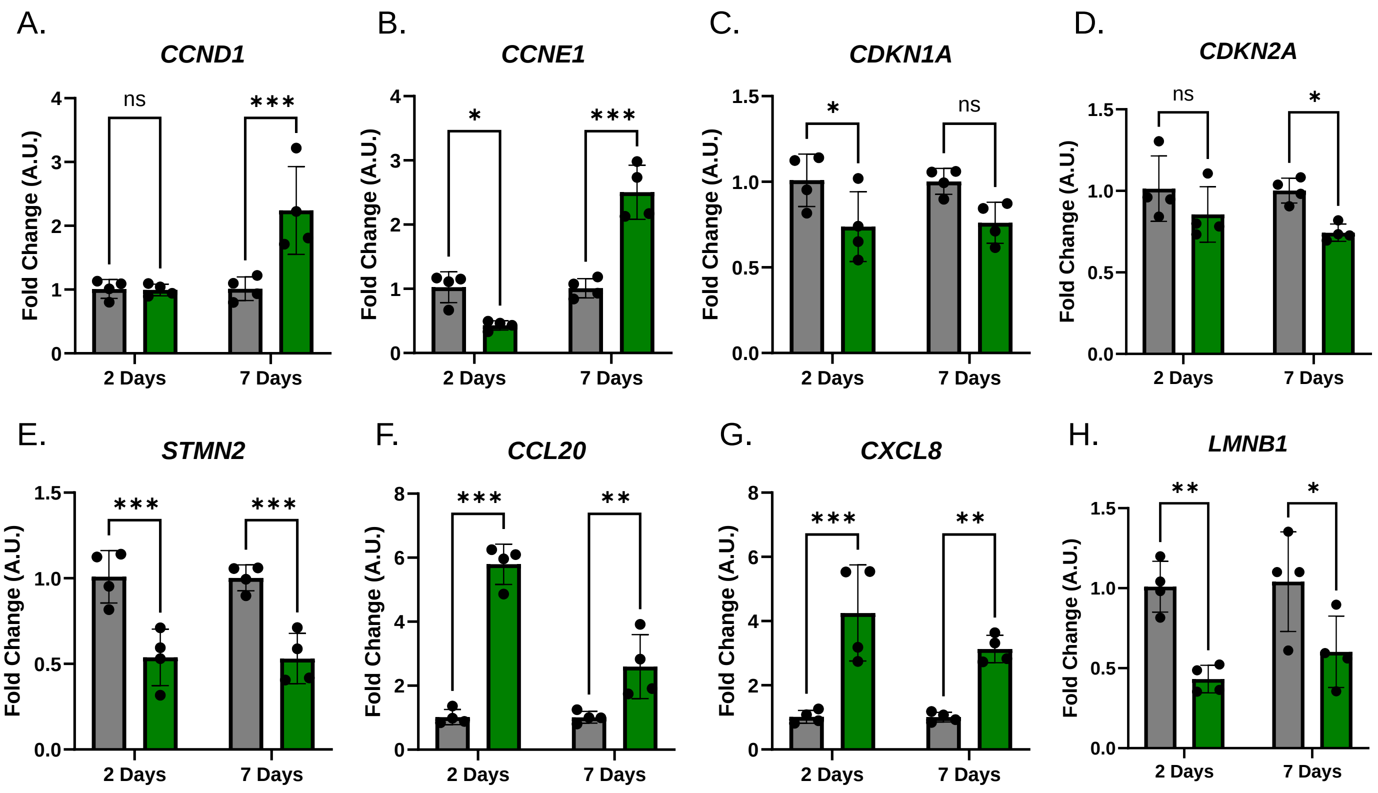
A.
CCND1
B.
CCNE1
C.
CDKN1A
D.
CDKN2A
E.
STMN2
F.
CCL20
G.
CXCL8
H.
LMNB1
Fold Change (A.U.)
2 Days
7 Days
ns
*
**
***

Figure S3

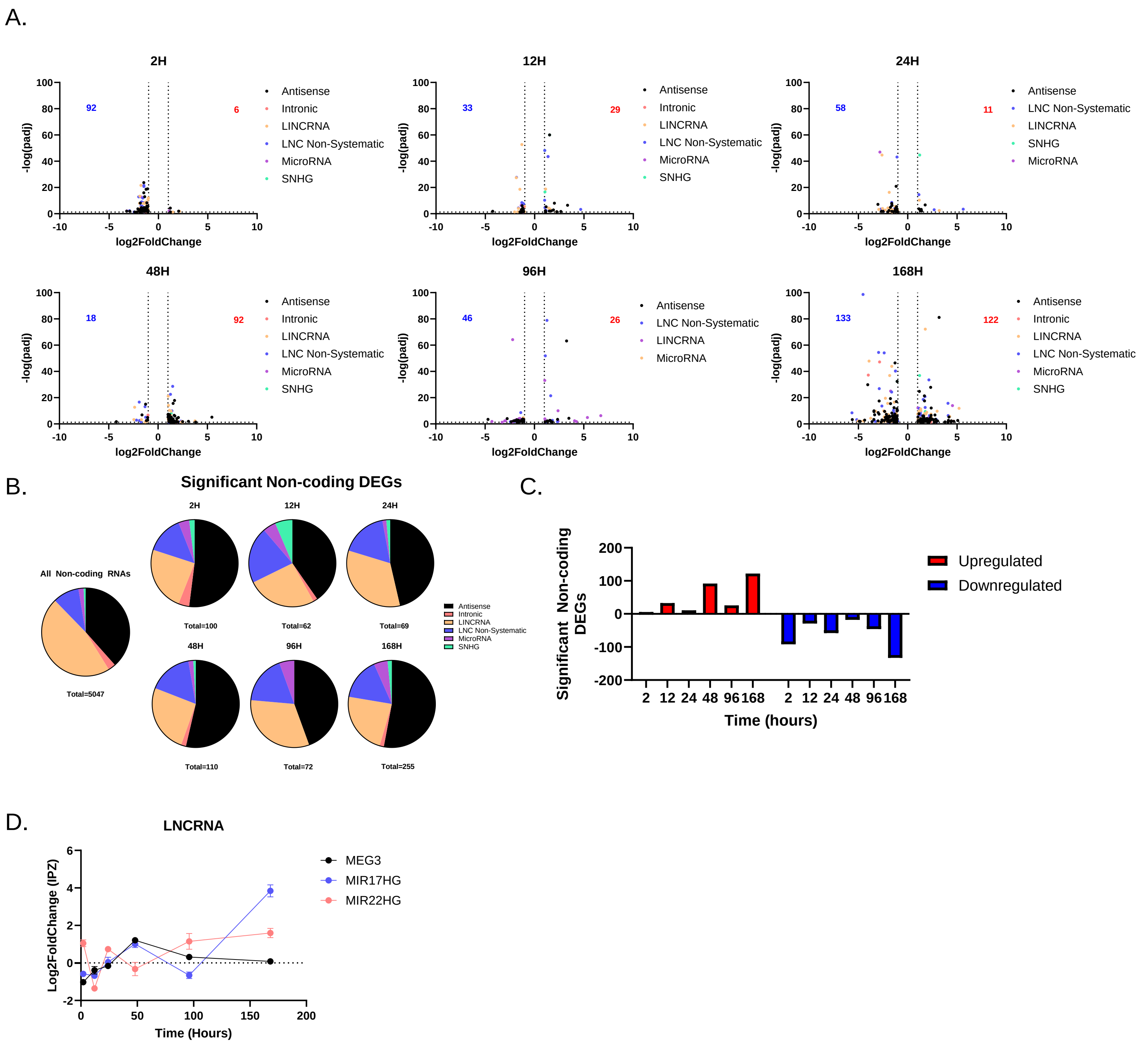
A.
2H
12H
24H
48H
96H
168H
-log(padj)
log2FoldChange
Antisense
Intronic
LINCRNA
LNC Non-Systematic
MicroRNA
SNHG
92
6
33
29
58
11
18
92
46
26
133
122
B.
Significant Non-coding DEGs
All Non-coding RNAs
Total=5047
Total=100
Total=62
Total=69
Total=110
Total=72
Total=255
C.
Significant Non-coding DEGs
Time (hours)
Upregulated
Downregulated
D.
LNCRNA
Log2FoldChange (IPZ)
Time (Hours)
MEG3
MIR17HG
MIR22HG

Figure S4

A.

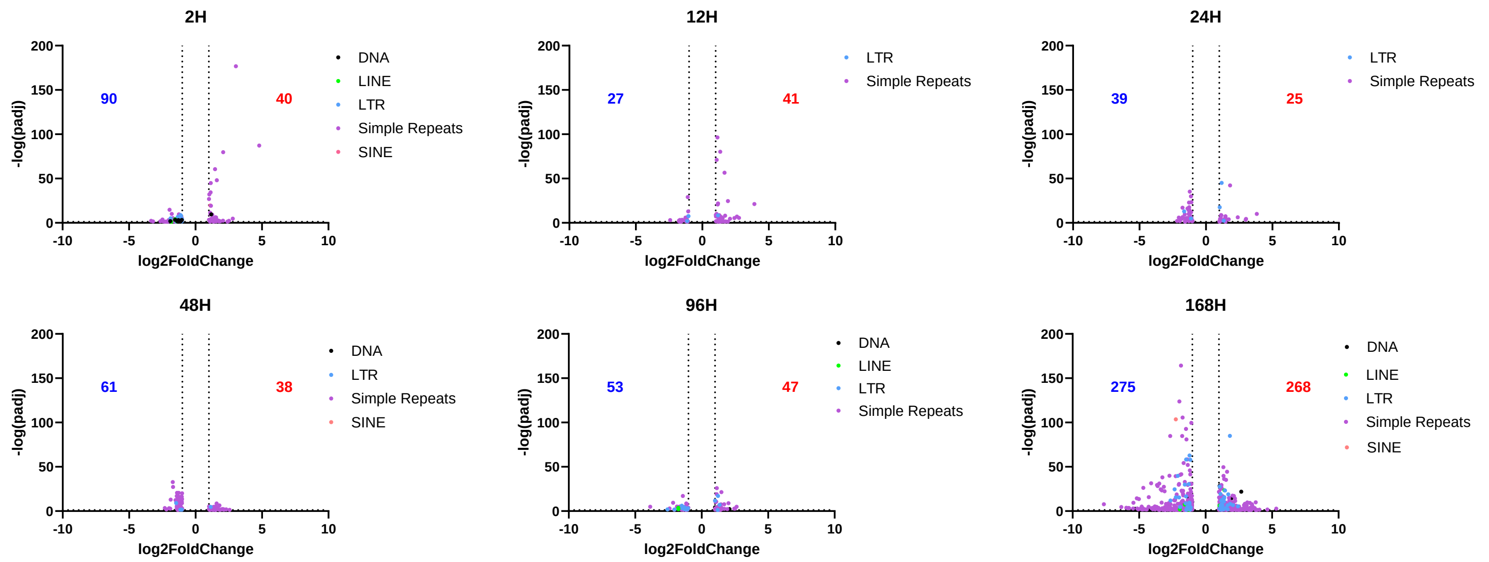

B.

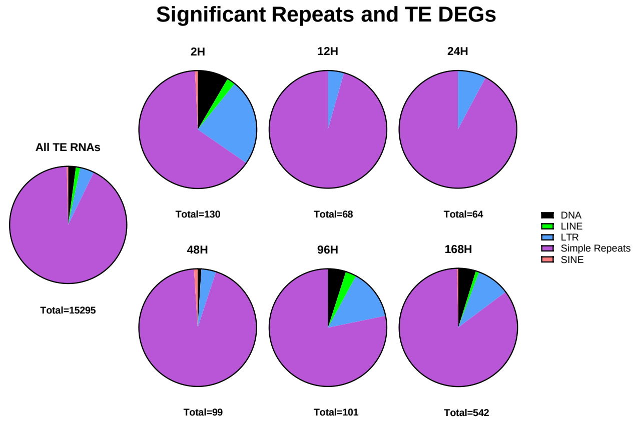

C.

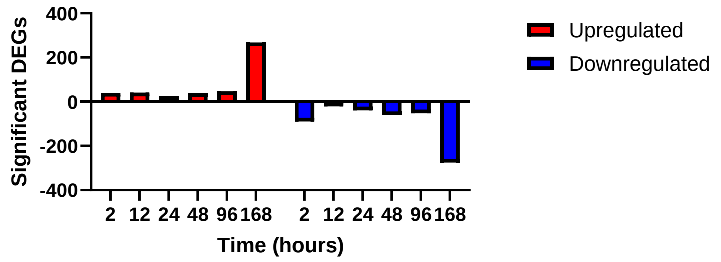

Figure S5

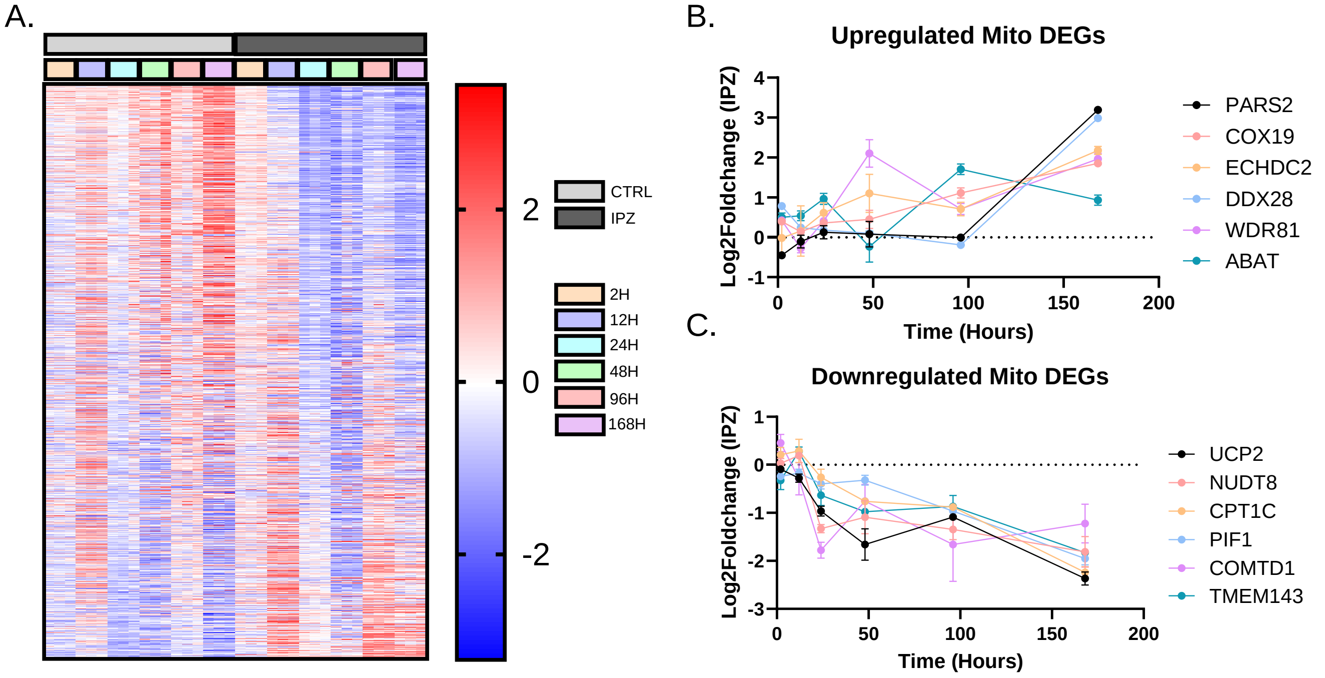

A.
CTRL
IPZ
2H
12H
24H
48H
96H
168H
2
0
-2
B.
Upregulated Mito DEGs
Log2Foldchange (IPZ)
Time (Hours)
PARS2
COX19
ECHDC2
DDX28
WDR81
ABAT
C.
Downregulated Mito DEGs
Log2Foldchange (IPZ)
Time (Hours)
UCP2
NUDT8
CPT1C
PIF1
COMTD1
TMEM143

Figure S6

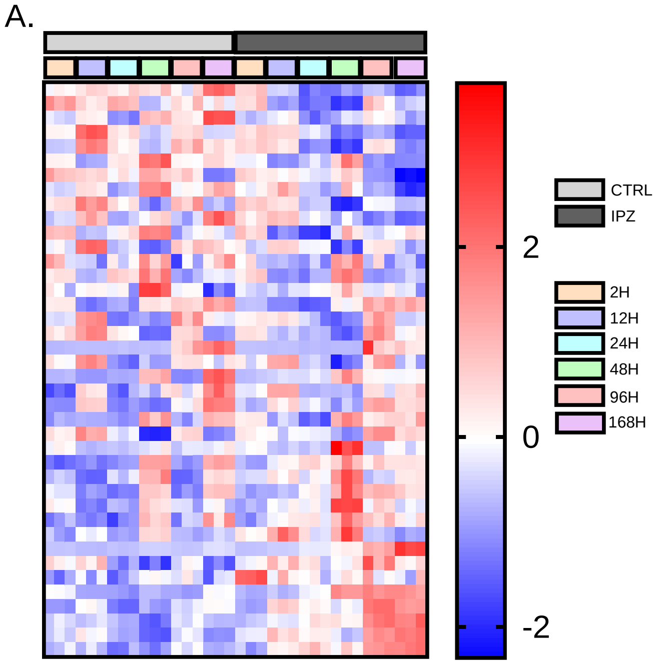
A.
2
0
-2
CTRL
IPZ
2H
12H
24H
48H
96H
168H

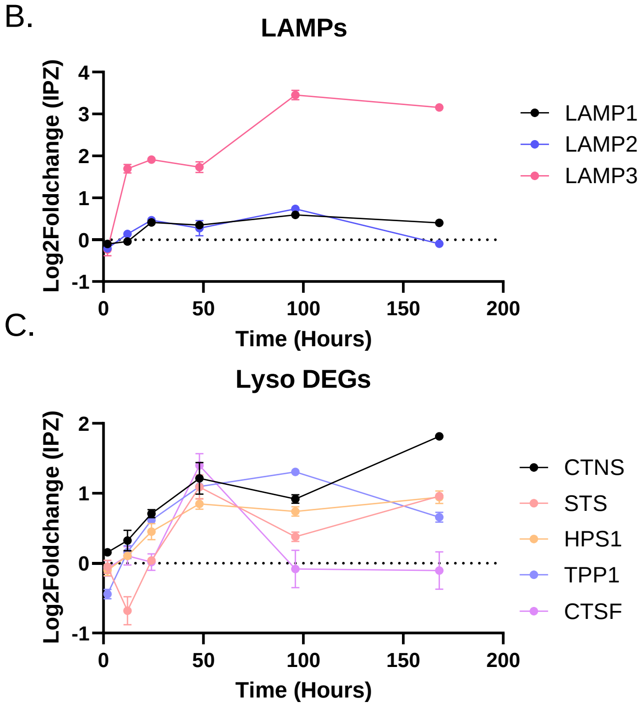
B.
LAMPs
Log2Foldchange (IPZ)
Time (Hours)
LAMP1
LAMP2
LAMP3
C.
Lyso DEGs
Log2Foldchange (IPZ)
Time (Hours)
CTNS
STS
HPS1
TPP1
CTSF

Figure S7

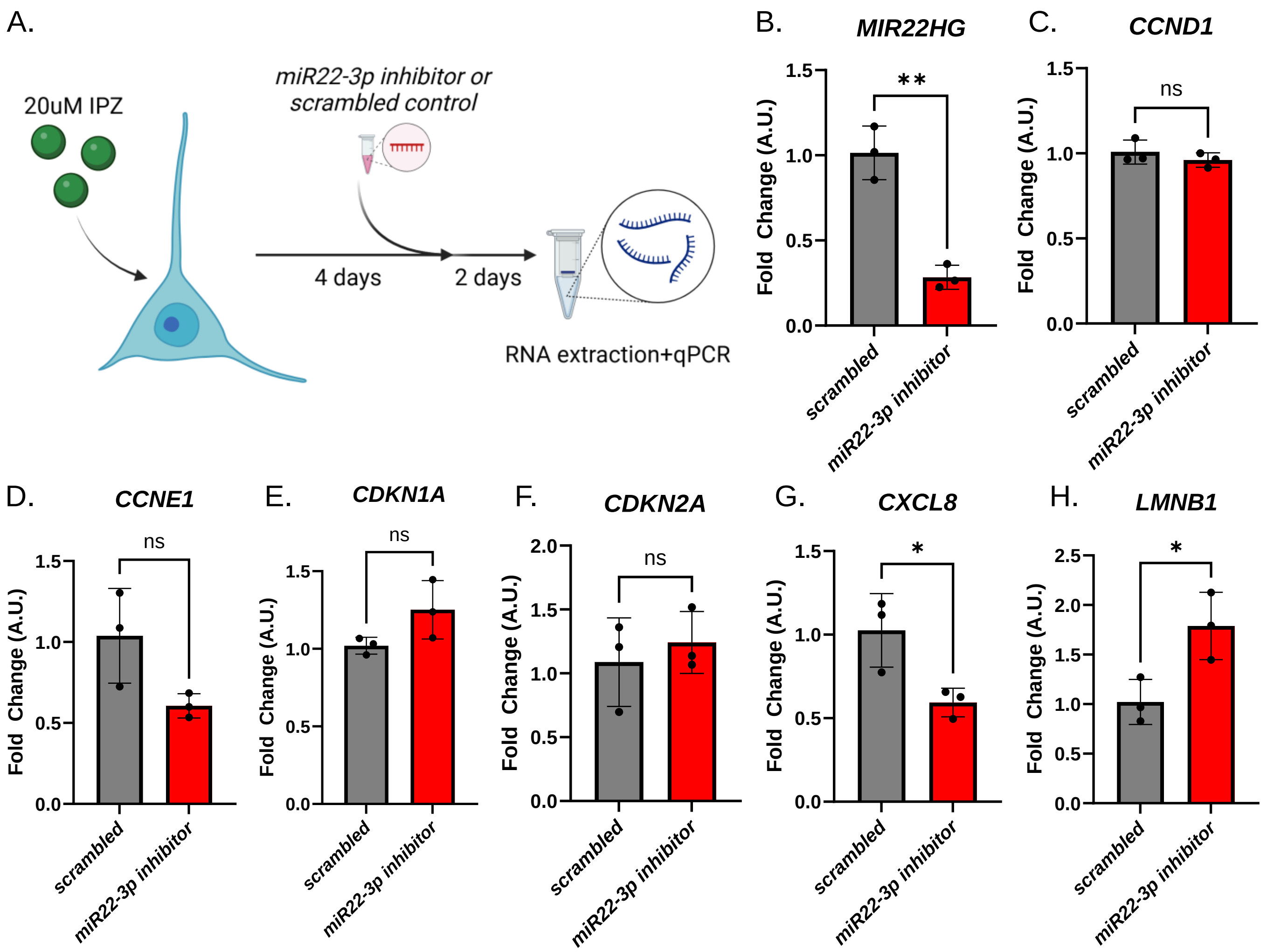

A.
20uM IPZ
miR22-3p inhibitor or scrambled control
4 days
2 days
RNA extraction+qPCR
B.
MIR22HG
Fold Change (A.U.)
**
scrambled
miR22-3p inhibitor
C.
CCND1
ns
D.
CCNE1
ns
E.
CDKN1A
ns
F.
CDKN2A
ns
G.
CXCL8
*
H.
LMNB1
*

Figure S8

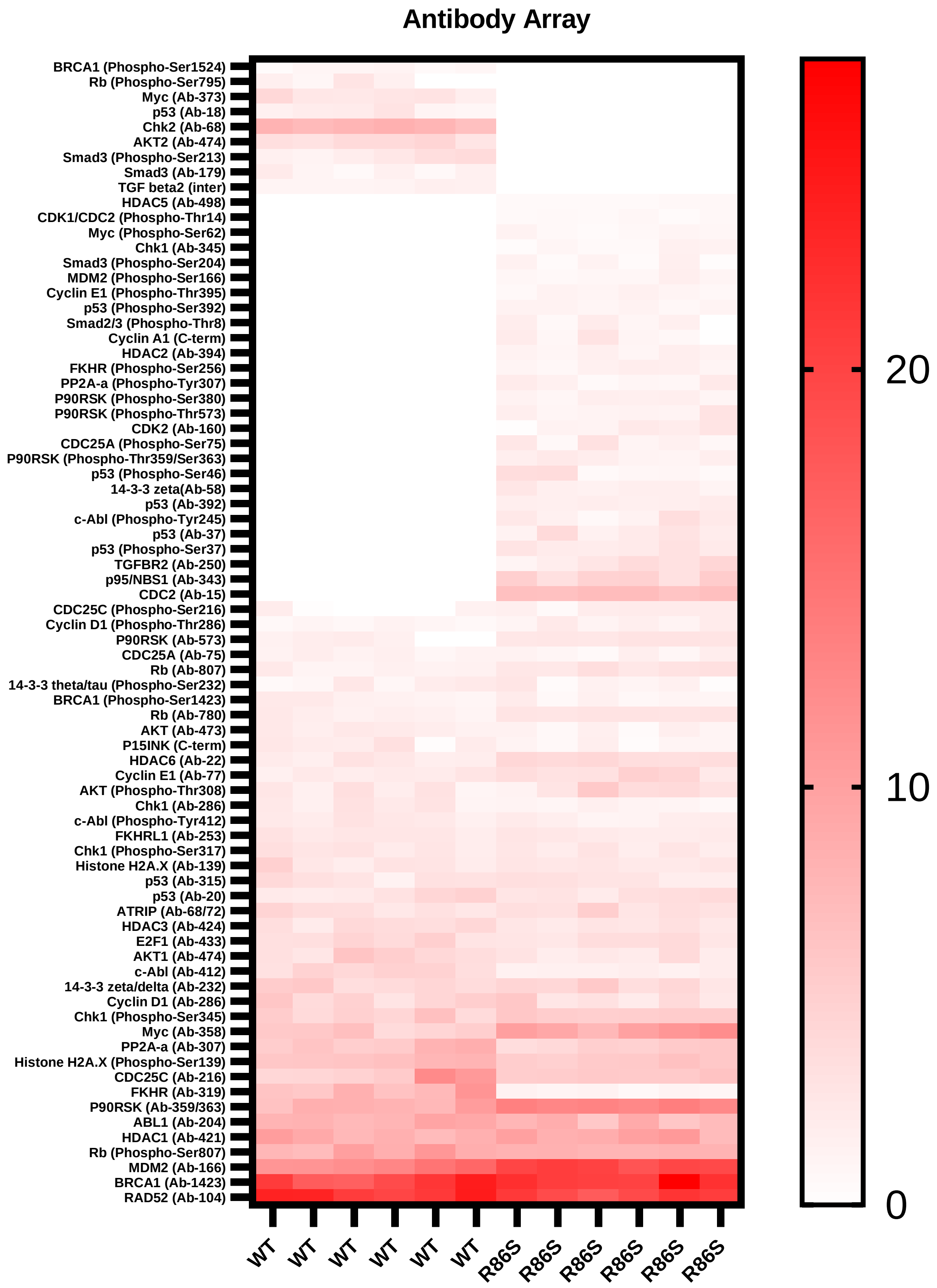

Antibody Array
BRCA1 (Phospho-Ser1524)
Rb (Phospho-Ser795)
Myc (Ab-373)
p53 (Ab-18)
Chk2 (Ab-68)
AKT2 (Ab-474)
Smad3 (Phospho-Ser213)
Smad3 (Ab-179)
TGF beta2 (inter)
HDAC5 (Ab-498)
CDK1/CDC2 (Phospho-Thr14)
Myc (Phospho-Ser62)
Chk1 (Ab-345)
Smad3 (Phospho-Ser204)
MDM2 (Phospho-Ser166)
Cyclin E1 (Phospho-Thr395)
p53 (Phospho-Ser392)
Smad2/3 (Phospho-Thr8)
Cyclin A1 (C-term)
HDAC2 (Ab-394)
FKHR (Phospho-Ser256)
PP2A-a (Phospho-Tyr307)
P90RSK (Phospho-Ser380)
P90RSK (Phospho-Thr573)
CDK2 (Ab-160)
CDC25A (Phospho-Ser75)
P90RSK (Phospho-Thr359/Ser363)
p53 (Phospho-Ser46)
14-3-3 zeta(Ab-58)
p53 (Ab-392)
c-Abl (Phospho-Tyr245)
p53 (Ab-37)
p53 (Phospho-Ser37)
TGFBR2 (Ab-250)
p95/NBS1 (Ab-343)
CDC2 (Ab-15)
CDC25C (Phospho-Ser216)
Cyclin D1 (Phospho-Thr286)
P90RSK (Ab-573)
CDC25A (Ab-75)
Rb (Ab-807)
14-3-3 theta/tau (Phospho-Ser232)
BRCA1 (Phospho-Ser1423)
Rb (Ab-780)
AKT (Ab-473)
P15INK (C-term)
HDAC6 (Ab-22)
Cyclin E1 (Ab-77)
AKT (Phospho-Thr308)
Chk1 (Ab-286)
c-Abl (Phospho-Tyr412)
FKHRL1 (Ab-253)
Chk1 (Phospho-Ser317)
Histone H2A.X (Ab-139)
p53 (Ab-315)
p53 (Ab-20)
ATRIP (Ab-68/72)
HDAC3 (Ab-424)
E2F1 (Ab-433)
AKT1 (Ab-474)
c-Abl (Ab-412)
14-3-3 zeta/delta (Ab-232)
Cyclin D1 (Ab-286)
Chk1 (Phospho-Ser345)
Myc (Ab-358)
PP2A-a (Ab-307)
Histone H2A.X (Phospho-Ser139)
CDC25C (Ab-216)
FKHR (Ab-319)
P90RSK (Ab-359/363)
ABL1 (Ab-204)
HDAC1 (Ab-421)
Rb (Phospho-Ser807)
MDM2 (Ab-166)
BRCA1 (Ab-1423)
RAD52 (Ab-104)
WT
WT
WT
WT
WT
WT
R86S
R86S
R86S
R86S
R86S
R86S
20
10
0

Table S1

Cell Culture	Company	Cat #
Dulbecoo's Modified Eagle's Medium	Corning	10-017-CV
Eagle's Minimum Essential Medium	ATCC	30-2003
FBS 1X	Gibco	26140079
Penstrep 100X	Gibco	15140122
Glutamax 100X	Gibco	35050061
Non-essential Amino-Acids 100X	Gibco	11140050
Trypsin-EDTA (0.05%)	Gibco	25300062
Hibernate-A	Gibco	A1247501
Neurobasal-A	Gibco	10888022
B27	Gibco	17504044
Antibody	Company	Cat #
MAP2	AVES	4H5
p16INK4a	Invitrogen	PA5-20379
p21Cip1	Invitrogen	MA5-31479
p27Kip1	Abcam	ab32034
Lamin B1	Abcam	Ab16048
yH2AX (Ser139)	Cell Signaling	20E3
Alexa Fluor 594 g@ms IgG2a	Invitrogen	A21135
Alexa Fluor 488 g@Rb Ig	Invitrogen	A11034
Alexa Fluor 488 g@Chk IgY	Invitrogen	A11039
Alexa Fluor 594 g@Chk IgY	Invitrogen	A11042
Alexa Fluor 594 g@ms IgG1	Invitrogen	A21125
Alexa Fluor 594 g@Rb Ig	Invitrogen	A11037
Alexa Fluor 594 g@ms IgG2b	Invitrogen	A21141
Alexa Fluor 405 g@Rb Ig	Invitrogen	A31556
IRDye 800CW Goat anti-Mouse IgG1	Licor	926-32350
IRDye 800CW Goat anti-Rabbit IgG	Licor	926-32211

Table S2

Primer	Forward	Reverse
Gapdh (mouse)	GGCAAATTCAACGGCACAGT	GGGTCTCGCTCCTGGAAGAT
Stmn2 (mouse)	TGTCACTGATCTGCTCCTGC	TGGGAGATGGTGGCTTCAAG
Gapdh (human)	GTCTCCTCTGACTTCAACAGCG	ACCACCCTGTTGCTGTAGCCAA
Actb (mouse)	CATTGCTGACAGGATGCAGAAGG	TGCTGGAAGGTGGACAGTGAGG
Cdkn1a (mouse)	TCGCTGTCTTGCACTCTGGTGT	CCAATCTGCGCTTGGAGTGATAG
Cdkn2a (mouse)	TGTTGAGGCTAGAGAGGATCTTG	CGAATCTGCACCGTAGTTGAGC
Cxcl8 (mouse)	CCTTTCCACCCCAAATTTAT	AAACTTCTCCACAACCCTCTG
Il6 (mouse)	TACCACTTCACAAGTCGGAGGC	CTGCAAGTGCATCATCGTTGTTC
E2f1 (mouse)	GGATCTGGAGACTGACCATCAG	GGTTTCATAGCGTGACTTCTCCC
Lmnb1 (mouse)	AGGAAGAGCTGGAGCAGACCTA	GCAGGTTAGAGAGCTGTGAGGA
Meg3 (mouse)	CACAGAAGACGAAGAGCTGGA	GGTAGAGGTGCACAGCAGGT
GAPDH (human)	GTCTCCTCTGACTTCAACAGCG	ACCACCCTGTTGCTGTAGCCAA
B-ACTIN (human)	CACCATTGGCAATGAGCGGTTC	AGGTCTTTGCGGATGTCCACGT
CDKN1A (human)	AGGTGGACCTGGAGACTCTCAG	TCCTCTTGGAGAAGATCAGCCG
CDKN2A (human)	CAACGCACCGAATAGTTACG	CTGCCCATCATCATGACCTGG
E2F1 (human)	GGATCTGGAGACTGACCATCAG	GGTTTCATAGCGTGACTTCTCCC
CXCL8 (human)	GAGAGTGATTGAGAGTGGACCAC	CACAACCCTCTGCACCCAGTTT
CCL20 (human)	AAGTTGTCTGTGTGCGCAAATCC	CCATTCCAGAAAAGCCACAGTTTT
LMNB1 (human)	GAGAGCAACATGATGCCCAAGTG	GTTCTTCCCTGGCACTGTTGAC
MEG3 (human)	GCATTAAGCCCTGACCTTTG	TCCAGTTTGCTAGCAGGTGA
CCND1 (human)	TCTGGCATTTTGGAGAGGAAGTG	TCTACACCGACAACTCCATCCG
CCNE1 (human)	TGTGTCCTGGATGTTGACTGCC	CTCTATGTCGCACCACTGATACC
MIR22HG (human)	CCAGTTGAAGAACTGTTGCCC	CGTATCATCCACCCTGCTGT
